# Trophic amplification: A model intercomparison of climate driven changes in marine food webs

**DOI:** 10.1371/journal.pone.0287570

**Published:** 2023-08-23

**Authors:** Vianney Guibourd de Luzinais, Hubert du Pontavice, Gabriel Reygondeau, Nicolas Barrier, Julia L. Blanchard, Virginie Bornarel, Matthias Büchner, William W. L. Cheung, Tyler D. Eddy, Jason D. Everett, Jerome Guiet, Cheryl S. Harrison, Olivier Maury, Camilla Novaglio, Colleen M. Petrik, Jeroen Steenbeek, Derek P. Tittensor, Didier Gascuel

**Affiliations:** 1 UMR Dynamics and Sustainability of Ecosystems: From Source to Sea (DECOD), Institut Agro, Ifremer, INRAE, Rennes, France; 2 Institute for the Oceans and Fisheries, The University of British Columbia, Vancouver, British Columbia, Canada; 3 Program in Atmospheric and Oceanic Sciences, Princeton University, Princeton, NJ, United States of America; 4 MARBEC, Univ. Montpellier, CNRS, Ifremer, IRD, Sète, France; 5 Institute of Marine and Antarctic Studies, University of Tasmania, Hobart, TAS, Australia; 6 Centre for Marine Socioecology, University of Tasmania, Hobart, TAS, Australia; 7 Potsdam-Institute for Climate Impact Research (PIK), Potsdam, Germany; 8 Centre for Fisheries Ecosystems Research, Fisheries & Marine Institute, Memorial University, St. John’s, NL, Canada; 9 School of Earth and Environmental Sciences, University of Queensland, Brisbane, QLD, Australia; 10 Centre for Marine Science and Innovation, School of Biological, Earth and Environmental Sciences, University of New South Wales, Sydney, NSW, Australia; 11 Commonwealth Scientific and Industrial Research Organisation (CSIRO) Environment, Queensland Biosciences Precinct, St Lucia, QLD, Australia; 12 Department of Atmospheric and Oceanic Sciences, University of California, Los Angeles, CA, United States of America; 13 Department of Coastal and Ocean Science and Center for Computation and Technology, Louisiana State University, Baton Rouge, LA, United States of America; 14 Scripps Institution of Oceanography, University of California San Diego, La Jolla, CA, United States of America; 15 Ecopath International Initiative, Barcelona, Spain; 16 Department of Biology, Dalhousie University, Halifax, NS, Canada; MARE – Marine and Environmental Sciences Centre, PORTUGAL

## Abstract

Marine animal biomass is expected to decrease in the 21st century due to climate driven changes in ocean environmental conditions. Previous studies suggest that the magnitude of the decline in primary production on apex predators could be amplified through the trophodynamics of marine food webs, leading to larger decreases in the biomass of predators relative to the decrease in primary production, a mechanism called trophic amplification. We compared relative changes in producer and consumer biomass or production in the global ocean to assess the extent of trophic amplification. We used simulations from nine marine ecosystem models (MEMs) from the Fisheries and Marine Ecosystem Models Intercomparison Project forced by two Earth System Models under the high greenhouse gas emissions Shared Socioeconomic Pathways (SSP5-8.5) and a scenario of no fishing. Globally, total consumer biomass is projected to decrease by 16.7 ± 9.5% more than net primary production (NPP) by 2090–2099 relative to 1995–2014, with substantial variations among MEMs and regions. Total consumer biomass is projected to decrease almost everywhere in the ocean (80% of the world’s oceans) in the model ensemble. In 40% of the world’s oceans, consumer biomass was projected to decrease more than NPP. Additionally, in another 36% of the world’s oceans consumer biomass is expected to decrease even as projected NPP increases. By analysing the biomass response within food webs in available MEMs, we found that model parameters and structures contributed to more complex responses than a consistent amplification of climate impacts of higher trophic levels. Our study provides additional insights into the ecological mechanisms that will impact marine ecosystems, thereby informing model and scenario development.

## 1. Introduction

Greenhouse gas emissions from anthropogenic activities have disrupted the natural carbon cycle [1], impacting all ecological compartments of the biosphere [2]. As a result of these changes in physical and chemical properties, major changes have already been observed at the base of the food web and are expected to be amplified in the coming decades, often negatively, at higher trophic levels—a mechanism known as trophic amplification [2–5].

Phytoplankton, which account for 90% of net primary production (NPP) and fuel marine food webs [6, 7], are affected by climate-driven changes in biomass and productivity through changes in nutrient availability, light limitation and thermal stratification of the water column [8]. Earth System Models (ESMs) within the 6th Coupled Model Intercomparison Project (CMIP6; [9]) project an overall global decrease of 3.0 ± 9.1% in NPP by the end of the 21^st^ century [10–13]. However, the ESMs also project large spatial variability in NPP changes.

Ocean warming, deoxygenation and acidification affect the physiological functions of marine organisms, including modifications in body function, growth rates, maximum body size and reproductive rates [4, 14–17]. Moreover, climate-induced changes in ocean conditions affect the biogeography and phenology of marine populations [2], consequently altering the trophodynamics of marine ecosystems [3, 18, 19]. These multiple climatic stressors may interact and amplify the impacts on marine ecosystems [4, 20, 21].

Changes in biomass and productivity at low trophic levels may be exacerbated at higher trophic levels through trophic amplification. This process refers to the bottom-up propagation of the climate signal from primary producers along the food web, with an increasing magnitude of impacts at each higher trophic level [3, 22–25]. Trophic amplification between phytoplankton and zooplankton has been demonstrated in simulations from different planktonic food web models and ESMs [22, 24, 26], with changes in zooplankton biomass around 2 times greater than those of phytoplankton.

Trophic amplification has also been explored at higher trophic levels, with regional-scale studies showing that indirect effects of increased temperature can be exacerbated through the food web, leading to greater impacts on the biomass of higher trophic levels [15]. Lotze et al. (2019) analysed trophic amplification based on a model intercomparison project using six global FishMIP Marine Ecosystem Models (MEMs) forced by standardised outputs of two contrasting ESMs and four climate change scenarios. They defined trophic amplification by comparing relative changes in total consumer biomass (MEMs outputs) to relative changes in NPP and low trophic level biomass (ESM outputs). They showed that, by the end of the 21st century, the total consumer biomass was projected to decline more strongly than NPP, but with high levels of uncertainty.

The nature of temperature-dependence of biological rates plays a role in the transfer efficiency of biomass or energy from one trophic level to the next (or consumer in a more complex food-web) and MEMs represent the details of these processes differently [25, 27]. Through trophic amplification, high trophic level species are hypothesised to be even more impacted by climate change with high biomass losses. These species play a crucial role in the stability of food webs and ecosystems in general [28, 29] and are the main target of most fisheries worldwide [30, 31]. Because high trophic level species play key ecological and socio-economic roles, it is important to better understand the underlying mechanisms to face the challenges that the 21st century will pose, including biomass loss and food security.

In this study, we examine the spatial and temporal patterns of trophic amplification in the global ocean, initially based on the definition of Lotze et al. (2019). We consider nine MEMs that have been forced by two CMIP6 ESMs under the high emissions scenario (SSP5-8.5) to investigate the trophic amplification process across a variety of marine ecosystem models with different assumptions, structures, and processes. We analyse how climate-induced NPP changes (ESM outputs) may propagate to higher trophic levels and explore spatial variations across the global ocean using a classification of biomass responses (MEMs outputs) adapted from Chust et al. (2014). Furthermore, we investigate the potential warming effects on the projected trophic amplification. Finally, we track trophic amplification through the food web, from primary consumer to high trophic levels, using all available MEMs outputs.

## 2. Materials and methods

### 2.1 Changes in biomass, primary productivity and temperature

This study analysed global projections from ESMs and MEMs available from CMIP6 and Fish-MIP, respectively [3, 5].

Outputs from two ESMs from CMIP6 were considered: IPSL-CM6A-LR developed by the Pierre Simon Laplace Institute (IPSL, [32]) and GFDL-ESM4 developed by the Geophysical Fluid Dynamics Laboratory (GFDL; [33]). ESMs integrate the interactions between the atmosphere, ocean, land, ice and biosphere to simulate the state of regional and global climate and their changes under a wide variety of greenhouse gas emissions pathways [10]. Greenhouse gas emissions pathways that are adopted by the Intergovernmental Panel on Climate Change (IPCC) are the Shared Socioeconomic Pathways (SSPs, [34]). Projections by ESMs under standardised simulation experiments and SSPs are available from Earth System Grid Federation (ESGF,https://esgf-node.llnl.gov/projects/esgf-llnl/).

CMIP6 ESMs data used to force FishMIP MEMs was processed to a two-dimensional horizontal regular 1° x 1° grid (https://data.isimip.org). Here we use this data on an annual time resolution from 1950 to 2099, under SSP5-8.5 where the concentration of greenhouse gases will rise throughout the 21st century. This choice of scenario has been elected in order to easily disentangle mechanisms within each MEMs and better showcase as well as understand models’ reaction to their forcing variables.

Outputs from nine MEMs were gathered from Fish-MIP (fishmip.org, [5], data extraction were made the 25 of November 2022): APECOSM, BiOeconomic mArine Trophic Size-spectrum model (BOATS), Dynamic Bioclimate Envelope Model (DBEM), Dynamic Benthic Pelagic Model (DPBM), EcoOcean, EcoTroph, FishErIes Size and functional TYpe model (FEISTY), Macroecological, and Zooplankton Model of Size Spectra (ZooMSS). MEMs were forced using a set of environmental outputs from the two ESMs based on a no-fishing and SSP5-8.5 scenario [3, 5, 27]. All MEMs used the IPSL outputs, while only seven MEMs used the GFDL outputs, due to specific and non-compatible data requirements of APECOSM and DBPM.

The nine selected MEMs differ in how they represent ecosystem structure, composition, and trophodynamics. For instance, ecosystem components are represented by size classes (BOATS and Macroecological), functional groups (EcoOcean), trophic levels (EcoTroph), species (DBEM), or a hybrid between size-classes and traits (APECOSM, DBPM, FEISTY, and ZooMSS), whereas food web links can be represented by trophic networks, diet composition or energy transfer ([35]; Tables 1 and S1). Each MEM is characterised by separate ecological assumptions to model the interactions with the environment and their ecological responses to changing environmental conditions [19].

**Table 1:** Functioning summary of the 9 MEMs used in the study. For more details on MEMs’ taxonomic scope, key features and drivers see S1 Table (modified from Heneghan et al., 2021; Lotze et al., 2019; Tittensor et al., 2021).

MEMs model	Brief model description	Taxonomic scope
**APECOSM** [36]	A 3-D dynamic energy budget based Eulerian model of size-structured marine populations and communities based on environmentally driven individual bio-energetics, trophic interactions, and behaviours that are upscaled to populations and communities.	Generic size-based communities are explicit (typically epipelagic, migratory, mesopelagic, and bathypelagic) and focus species.
**BOATS** [37]	Sized-structured model that combines size-based ecological theory and metabolic constraints to estimate the production of fish biomass. This model resolves three size spectra for three separate species size groups.	All commercial species represented by three groups, defined in terms of the asymptotic mass.
**DBEM** [38]	DBEM defines a bioclimatic envelope (niche) for each species and simulates changes in growth, reproduction and population dynamics including abundance and carrying capacity under a varying environment.	Fish and invertebrate species (primarily commercial).
**Dynamic Benthic–Pelagic Model (DBPM)** [39]	A functional trait-based size-spectrum model that joins a pelagic predator size-spectrum with a benthic detritivore size spectrum; can include functional groups that do not feed according to size and unstructured resources.	Broadly represents “pelagic” fish predators and “benthic” invertebrates but can include herbivorous fish; flexible functional groups.
**EcoOcean** [40]	EcoOcean is a global food web model based on species interactions and transfer of energy across trophic levels (EwE framework). It is designed to evaluate the impact of climate change and human pressure on mobile marine ecosystems.	All trophic levels and taxonomic groups included as biomass pools (51 groups).
**EcoTroph** [27, 41, 42]	Global scale model representing the biomass flows from primary producers to top predators. Taking in account of metabolism to compute biomass by trophic level and can evaluate climate change impacts	Species are not resolved, only trophic level classes.
**FishErIes Size and functional TYpe model (FEISTY)** [43]	FEISTY is a spatially explicit, size- and trait-based model of higher trophic level dynamics based on first principles. It describes the three main commercially harvested fish functional types: forage fish, large pelagic fish, and demersal fish.	Forage, large pelagic and demersal fish, as well as benthic invertebrates, between 1 mg and 125 kg.
**Macroecological** [44]	A static model size-structure model, which uses minimal inputs together with ecological and metabolic scaling theory to predict mean size composition and abundance of animals (including fish)	180 body mass classes, Species are not resolved, only body mass classes.
**Zooplankton Model of Size Spectra (ZooMSS)** [45]	ZooMSS is a size- and trait-based model working at the functional group scale. It uses the functional size-spectrum framework to resolve the body size ranges, size-based feeding characteristics and carbon content of the nine most abundant zooplankton groups and three (small, medium and large) fish groups.	Nine zooplankton groups, from flagellates to jellyfish and all marine animals between 1 mg and 10 tonnes.

The responses of the MEMs that we considered are driven primarily by temperature and low trophic levels (primary production, phytoplankton and/or zooplankton concentrations), although oxygen, salinity, and ocean advection are considered in a subset of models (APECOSM, DBEM) and play a secondary role [19, 46, 47]. The projected changes in lower trophic level forcing were considered in all these MEMs by integrating changes in phytoplankton concentration, zooplankton concentration, seafloor particulate organic matter, and/or depth-integrated NPP, all outputs of the ESMs. In contrast, temperature effects throughout the food web were integrated differently among the MEMs. For example, in EcoTroph, temperature directly influences the transfer efficiency [27, 42], which is the fraction of energy transferred from one trophic level (TL) to the next, thus summarising losses in the food web [18]. In APECOSM, BOATS, DBPM, EcoOcean, FEISTY, Macroecological, and ZooMSS, temperature directly influences mortality and growth rates [19, 44]. Temperature impacts the fraction of NPP available to consumers in BOATS, which indirectly influences the length of the food chain [48]. In addition, APECOSM, DBPM, EcoOcean, FEISTY, and ZooMSS explicitly resolve predator-prey dynamics that scale with temperature (Tables 1 and S2)

The ecological outputs of each MEM on a two-dimensional horizontal regular 1° x 1° grid were gathered at a yearly time resolution from 1971 to 2099. The main output we compare is the estimated total consumer biomass, defined as the biomass of all organisms with trophic level >1.

**Fig 1:**
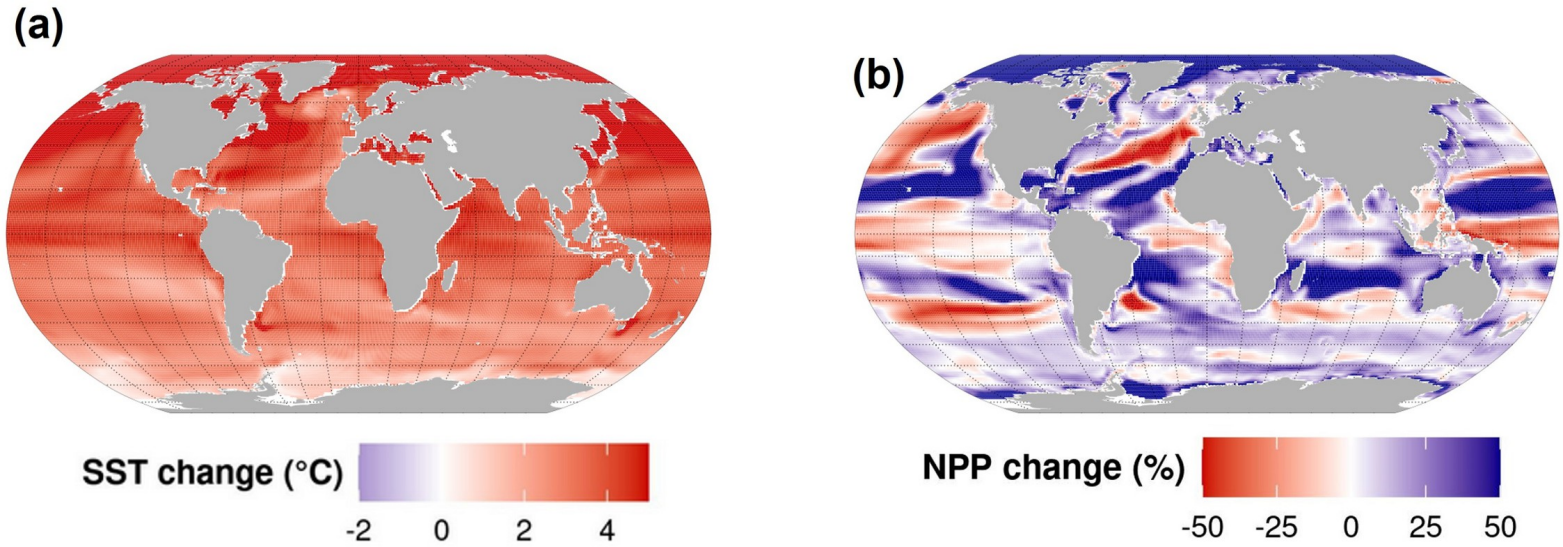
Main outputs of the Earth system models considered in the current study: mean change in Sea Surface Temperature (SST) (a) and percent change in Net Primary Production (NPP) (b) in the 2090s relative to the reference period 1995–2014, under SSP5-8.5 for GFDL.

In order to compare projections among MEMs and ESMs, we considered time series of global average percentage change in biomass and NPP, as well as spatial distributions of percentage change in biomass and NPP by grid cells between two reference decades (GFDL, Figs 1 and S1A, and IPSL, S1B and S2 Figs). For temporal trends, changes were calculated as annual percentage change relative to the 1995–2014 decade (the IPCC Sixth Assessment Report reference decade; [49]). For spatial distributions, changes were calculated as percentage change between the 1995–2014 time period and 2090–2099, the last decade of projections. Further, we calculated changes in SST as the absolute difference between the global annual average and the reference decade for temporal trends, and between the 2090–2099 time period and the reference decade for spatial changes (Fig 1).

### 2.2 Biomass response types

**Fig 2:**
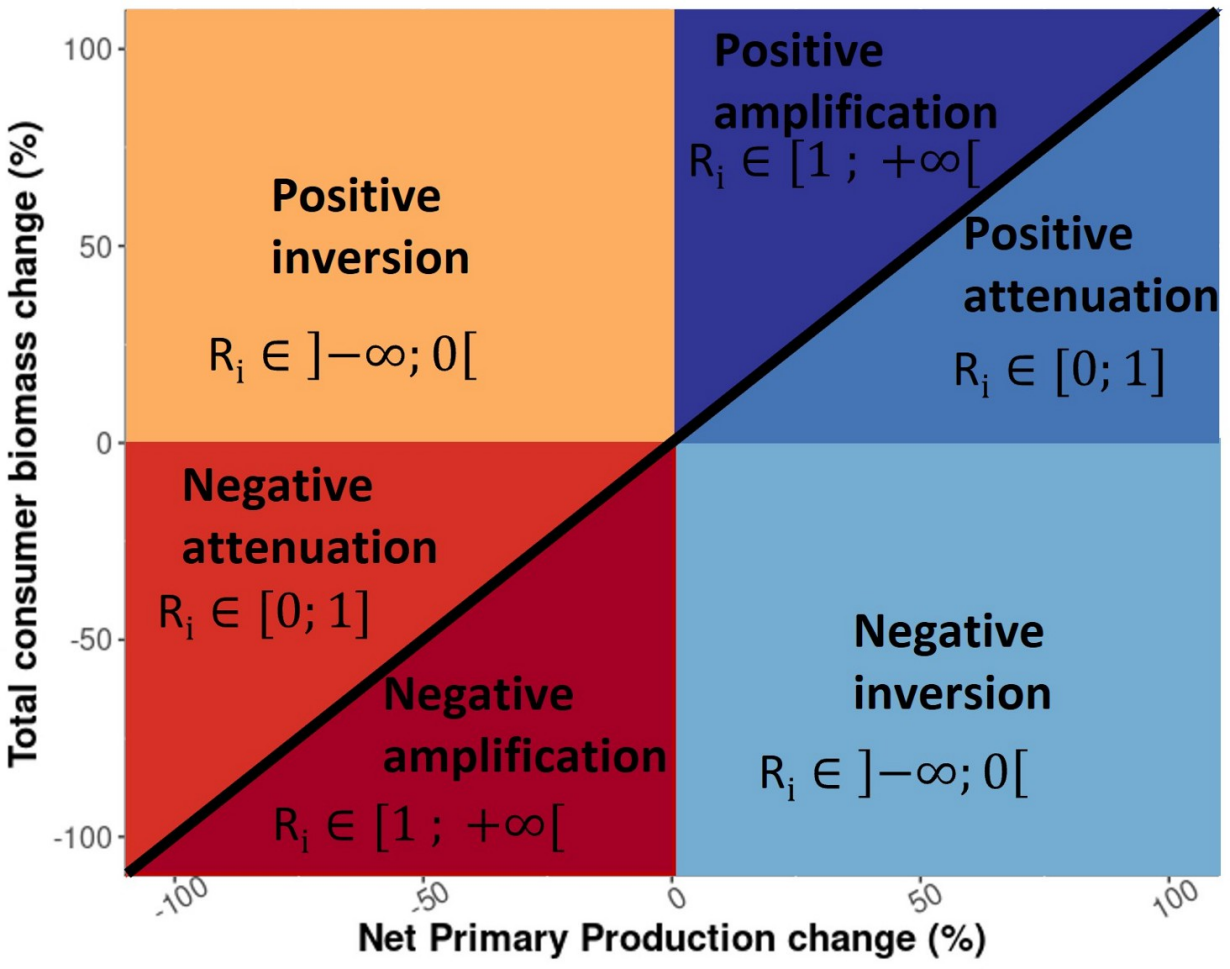
Conceptual scheme showing the six total consumer biomass response types to changes in NPP. (For interpretation of the references to colour in this figure legend, the reader is referred to the web version of this article).

The cascading effects of changes in NPP on upper trophic level biomass along the food web simulated by the ESMs and MEMs were investigated. The characteristics of such cascading effects were categorised into three types, according to the magnitude and direction of the impacts relative to the changes in NPP. The types of responses include amplification, attenuation, and inversion, in which the change in consumer biomass is relatively larger, smaller or in the opposite direction, respectively, compared to changes in NPP. Furthermore, the direction of changes in total consumer biomass (positive or negative) was added to qualify each type. Thus, a total of six consumer biomass response types associated with different values of change in NPP were examined (Fig 2) [22].

These six biomass response types were used to qualitatively compare the various MEMs and extract a common signal over all MEMs. We identified the 1° x 1° cells of the global ocean where the majority (7 out of 9 for IPSL and 6 out of 7 for GFDL) of the MEMs analysed in this study projected the same biomass response type by the 2090–2099 decade relative to the reference period, under SSP5-8.5 scenario.

### 2.3 Impact of warming on the biomass response to NPP change

We defined a metric, *R*_*i*_, to quantitatively measure trophic amplification. *R*_*i*_ is calculated by dividing the relative change in total consumer biomass by the relative change in NPP in each cell *i* between the 2090–2099 decade and the reference period:

$$R_{i}\;=\;\frac{Total\;consumer\;biomass\;change_{i}}{Net\;Primary\;Production\;change_{i}}$$

A value greater than 1 indicates an amplification of NPP changes; a value between 0 and 1 describes an attenuation of NPP changes and a negative value indicates an inversion (Fig 2).

Since several studies have suggested that trophic amplification arises directly or indirectly from temperature-dependent ecological processes [3, 15, 25, 27], we investigated the impact of SST on the metric *R*_*i*_. The objective was to test the hypothesis that a greater increase in SST would result in a greater trophic amplification and thus a greater *R*_*i*_. For each MEM and ESM, the ratio *R* is represented as a function of the SST increase relative to the reference period 1995–2014. Then, the *R*_*i*_ are averaged across all cells characterised by the same SST increase, regardless of the year. Because ecological processes at play could differ when NPP is expected to increase or decrease, two separate analyses were conducted, thus splitting the cells into two subsets of data depending on the direction of change of NPP. In addition, very low changes in NPP, between -1% and +1%, and extreme values of the *R* ratio, the 2.5 lowest and highest percentiles, were not considered in the analysis to avoid outliers. Finally statistical tests (Shapiro and Pearson) were implemented to assess variables’ dependence to each other.

### 2.4 Trophic amplification mechanisms in the food webs

First, to investigate how trophic amplification propagates through the food web and reaches higher trophic levels, we use all Fish-MIP data available and assess whether or not MEMs demonstrate trophic amplification through the food web. Of the models that provided biomass data by cutting food web segments, biomass values were available either by size class (EcoOcean, FEISTY), weight class (APECOSM, DBPM, BOATS, and Macroecological) or trophic class (EcoTroph). The biomass values expressed by trophic level in EcoTroph were converted from trophic classes to weight classes using the conversion S2 Table.

In a second step, in order to compare the outputs of each model, we standardised the values by calculating the relative change in biomass of each class compared to the reference period 1995–2014 similarly as for total consumer biomass:

We thus obtained relative consumer biomass change for 6 weight classes for APECOSM, DBPM, EcoTroph and Macroecological (1-10g, 10-100g, 100g-1kg, 1-10kg, 10-100kg, and >100kg), 4 weight classes for BOATS (10-100g, 100g-1kg, 1-10kg and 10-100kg), 3 length classes for EcoOcean (<30cm, [30-<90cm], > = 90cm), and 2 length classes for FEISTY (<30cm, > = 90cm).

Finally, in a complementary analysis (Supporting information and S8 Fig), we focused on EcoTroph FEISTY, and BOATS to explore and discuss two key aspects of trophic amplification in more detail: the trophic amplification of the production changes (instead of biomass changes), from primary producers to consumers, and the relationship between changes in consumer production and consumer biomass.

## 3. Results

### 3.1 Global trends of trophic amplification

**Fig 3:**
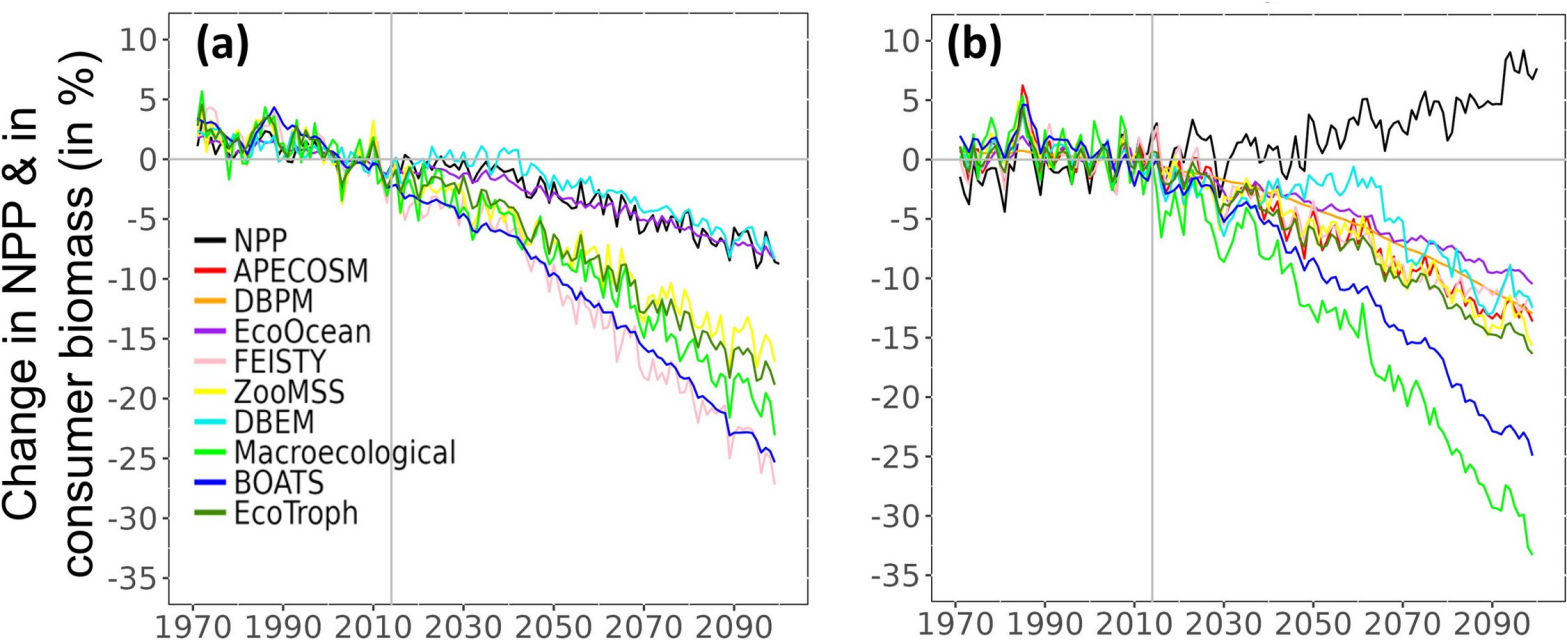
Ensemble projections of NPP and total consumer biomass changes, relative to 1995–2014, under SSP5-8.5 and for all the considered MEMs. (a) based on GFDL/MEMs combinations; (b) based on IPSL/MEMs combinations. All values are relative to the standardised reference period of 1995–2014. Vertical grey line indicates the last year of the historical period.

In CMIP6, GFDL and IPSL project a 7% decrease and a 7% increase in NPP and a 2 and 4°C increase in SST by the end of the century (2090–2099), respectively (Fig 3). On a global scale, mean projected total consumer biomass from the full MEM ensemble declines 9.6% more than NPP by the end of the century (2090–2099) under GFDL, while total consumer biomass and NPP showed opposite trends for IPSL (-16.0% and +7.6%, respectively) (Fig 3).

Projections based on GFDL showed a global amplification, with a larger decrease in total consumer biomass of 16.9%, 16.5%, 13.0%, 10.0%, and 8.0% for five MEMs (FEISTY, BOATS, Macroecological, EcoTroph, ZooMSS, respectively) compared to NPP projections, by the end of the 21st century. Amplification is barely observed in EcoOcean and DBEM, since they projected a drop in total consumer biomass similar to that in NPP (Fig 3A).

Under IPSL, MEM projections showed an inversion of the changes between NPP and total consumer biomass with major differences in magnitude. While the lower trophic level biomass driven models, EcoTroph, ZooMSS, APECOSM, DBPM, FEISTY, DBEM, and EcoOcean, projected decreases of 15.0%, 13.7%, 12.8%, 12.1%, 11.4%, 11.4%, and 9.7% in total consumer biomass, respectively, the lower trophic level production driven models, Macroecological and BOATS, projected much larger decreases of 30.0% and 23.3% in total consumer biomass, respectively (Fig 3B).

### 3.2 Different biomass response types

**Fig 4:**
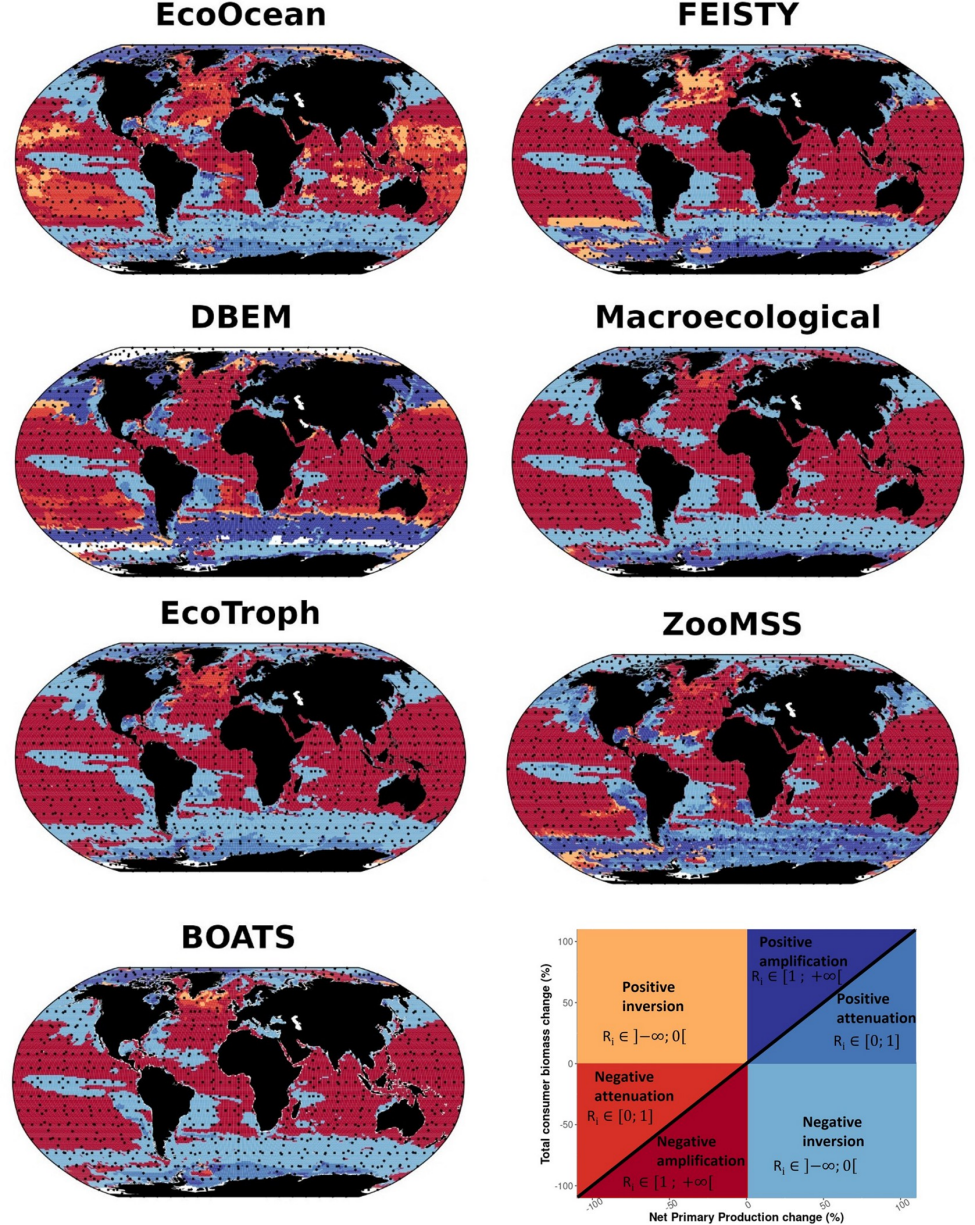
Spatial distribution of total consumer biomass response types, for the seven considered MEMs forced with GFDL-SSP5-8.5. Each response type is estimated at a 1° × 1° resolution, from the change in consumer biomass and NPP expected at the end of the century, relative to the reference period (Figs 1 and S1A).

MEM projections that are driven by GFDL reveal spatial contrasts in the responses of biomass compared to the climate-induced change in NPP (Fig 4). For all MEMs driven by GFDL, a decrease in total consumer biomass is projected over a large majority of the global ocean (86% ± 8% of total ocean area on average, S1A Fig). This decrease is mainly associated with negative amplification (in 55% of the global ocean on average over the seven MEMs), but also with negative inversion (27%) and negative attenuation (4%) (S3 Fig). Where NPP is projected to decrease (61% of the ocean surface), negative amplification is projected in 89% of these areas on average of the seven MEMs. In contrast, where NPP is projected to increase (39% of the ocean surface), a negative inversion is projected in 69% of the ocean waters, yet with 15% and 16% positive attenuation and positive amplification projected, respectively (S3 Fig).

Under IPSL forcing, total consumer biomass is projected to decrease by 76% ± 10.5% of the ocean surface (across nine MEMs). This biomass decrease is in majority dominated by a negative inversion response (46%, S4 Fig). Where NPP is projected to decrease (31% of the ocean surface), negative amplification is predominantly projected (78% ± 20% of the ocean surface). Otherwise, when NPP is projected to increase (69% of the ocean surface), similar contrasts between MEMs and qualitative results appeared, compared to GFDL (S4 and S5 Figs).

**Fig 5:**
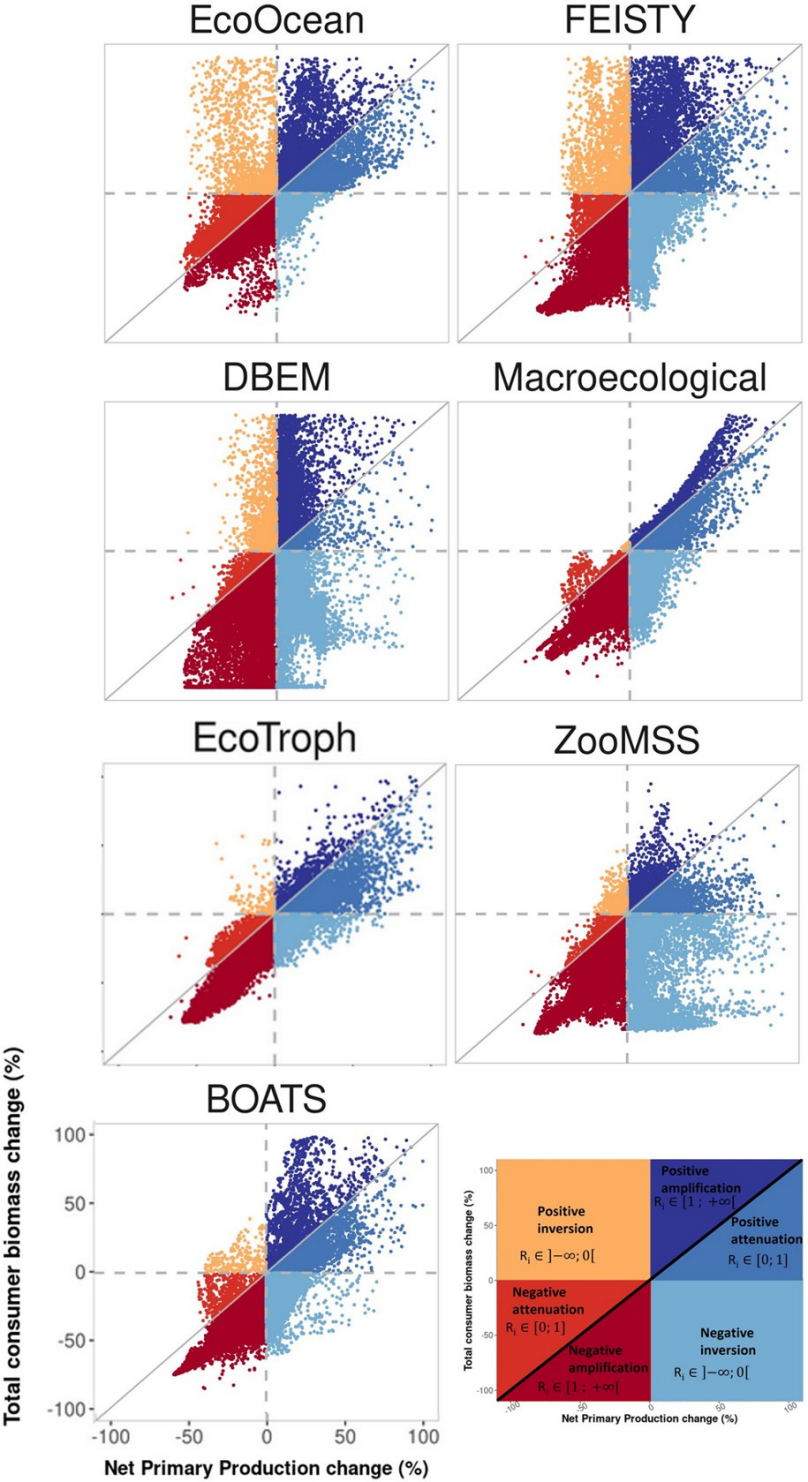
Magnitude of the different types of change for the seven considered MEMs forced with GFDL-SSP5-8.5. Each response type is estimated at a 1° × 1° resolution, from the change in consumer biomass and NPP expected at the end of the century, relative to the reference period (Figs 1 and S1A).

The magnitude of the biomass changes to NPP changes ratio (*R*) is dependent on biomass response types and MEMs (ANOVA, p_value<0.05), yet not on ESMs (ANOVA, p_value>0.05) (Figs 5 and S4B). Where an amplification response is projected, biomass decreases on average three times more than NPP decreases (Negative amplification, *R* = 3 ± 0.3) and increases two and a half more than NPP increases (Positive amplification, *R* = 2.5 ± 0.8). Where the NPP climate signal is negatively or positively attenuated through the food web, biomass decreases or increases by half of NPP (*R* = 0.5±0.1) in both cases. Where the NPP climate signal is inverted (negatively or positively), consumer biomass changes twice as much as NPP changes in the opposite direction (*R* = -2.2 ± 0.7). Finally, MEMs forced by lower trophic level biomass project lower magnitude *R* than lower trophic level production-forced MEMs (mainly DBPM and EcoOcean).

### 3.3 MEM agreement

**Fig 6:**
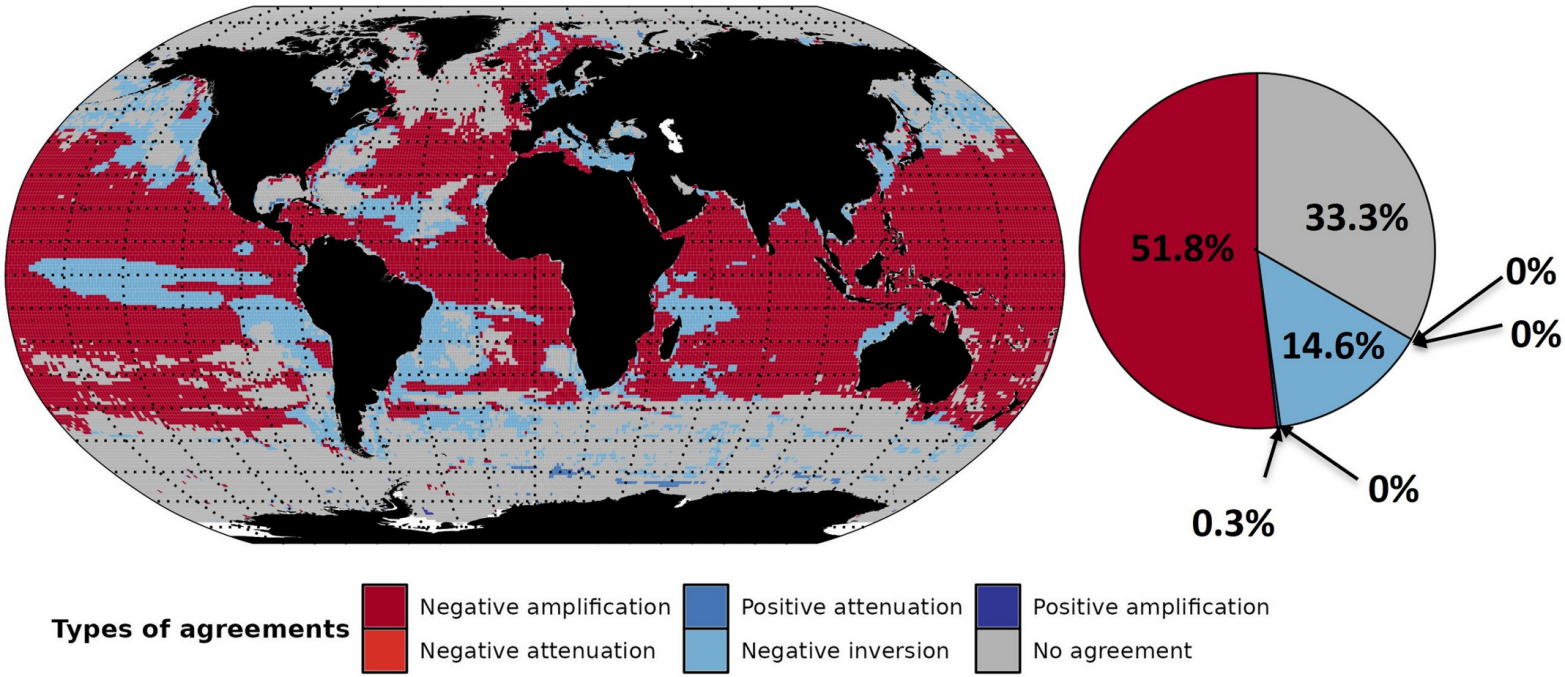
Model agreement for biomass response types projected in the 2090s, under GFDL-SSP5-8.5 configuration. Response types correspond to at least six out of seven models in agreement for the coloured cells, while grey cells indicate where fewer than six models project the same type of response and white cells where no data were available. The percentage numbers in the pie chart correspond to the relative surface areas.

With the GFDL forced SSP5-8.5 scenario, MEM projections strongly agree, *e*.*g*., exhibit the same biomass response type for at least six models, between 40°S and 50°N latitude. Disagreement among MEMs most often occurs in the polar regions (above 70°N and below 60°S under IPSL and GFDL forcing). Models are globally in agreement across the major parts of the ocean, in 66% and 70% of the ocean’s waters for GFDL (Fig 6) and IPSL (S6 Fig), respectively.

In particular, under GFDL forcing there is an agreement (Fig 6) for at least six models (FEISTY, ZooMSS, DBEM, Macroecological, BOATS, and EcoTroph) in the areas where biomass is projected to decrease (51.8% of negative amplification and 14.6% negative inversion). In contrast, biomass is projected to increase, with at least six models in agreement, for only 0.3% of the ocean surface. In other words, even where GFDL projects an increase in NPP (38.5% of the ocean), the biomass is generally projected to decrease.

Similarly, under IPSL, there is a strong agreement (64%) for at least seven models (APECOSM, FEISTY, ZooMSS, DBEM, Macroecological, BOATS, and EcoTroph) in the areas where biomass is projected to decrease with 38% and 25% of the ocean surface area corresponding to negative inversion and negative amplification, respectively (S6 Fig). Furthermore, models agree in 6% of the ocean surface, projecting an increase of total consumer biomass.

### 3.4 Warming effects on biomass responses to changes in NPP

**Fig 7:**
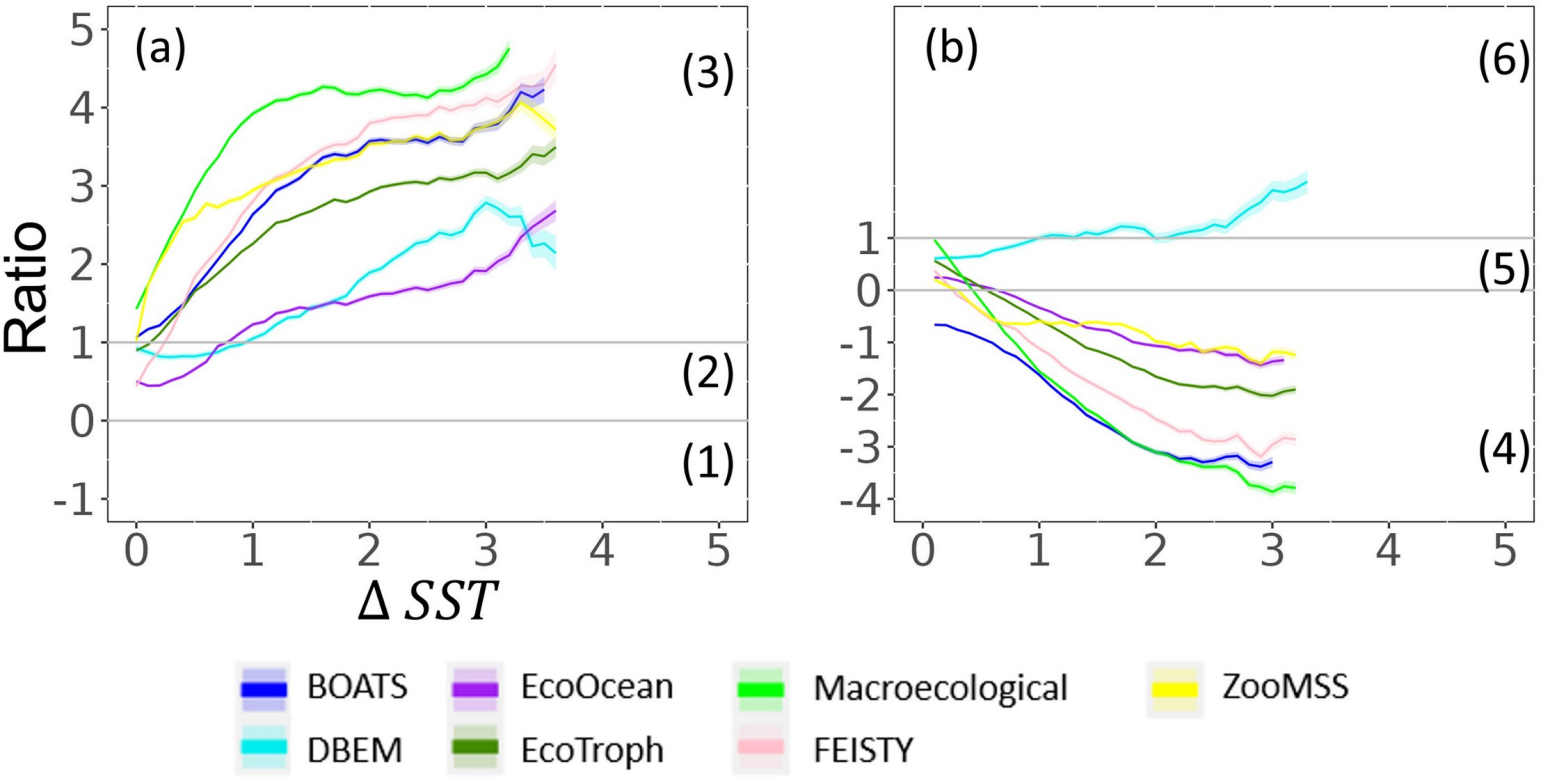
Evolution of *R* ratio (total consumer biomass change divided by NPP change) as a function of SST increase relative to the reference period: where NPP is expected to decrease (a) or increase (b) under GFDL-SSP5-8.5. Grey lines separate biomass response types. (1), (2) and (3) refer to positive inversion, negative attenuation, and negative amplification, respectively, while (4), (5) and (6) refer to negative inversion, positive attenuation, and positive amplification, respectively. Colour shaded areas correspond to standard deviation.

In the case of an expected decrease in NPP, biomass response types are correlated with the magnitude of warming under GFDL and IPSL (Pearson’s p-value<0.05, R = 0.79 ± 0.1 and R = 0.64 ± 0.4, respectively) (Figs 7A and S7A).

Under GFDL forcing, trophic amplification is observed for all MEMs, with a decrease in total consumer biomass larger than the decrease in NPP (and thus, *R*, the ratio of change is greater than 1). ZooMSS, Macroecological, BOATS and EcoTroph project trophic amplification even where no increase in SST is projected, and amplification increases as warming intensifies. Thus, the decrease in biomass for these models is about two and a half to four times that of NPP when SST increases by more than 2°C. In contrast, EcoOcean, FEISTY, and DBEM, project an attenuation of the NPP signal when SST does not change or increase a little (0<*R*<1, *ΔSST* = 0, Fig 7). But these three models also exhibit an increasing ratio of change according to the magnitude of warming, highlighting trophic amplification for all increases in SST larger than 0.8°C. Thus, warming appears to be a factor strengthening trophic amplification.

In the case of an expected increase in NPP (NPP change > 1%), a negative inversion (*R* < 0) is observed, with an effect of the warming intensity which offsets the increase in NPP and induces a decrease in total consumer biomass (Fig 7B). DBEM is an exception with a projected positive attenuation and positive amplification (*R*>1) in response to temperature increases.

The same analysis with IPSL, projecting a SST increasing up to 5°C compared to 3°C under GFDL, exhibits similar qualitative results (S7B Fig), except that only ZooMSS and Macroecological project negative amplification without SST increases, APECOSM, BOATS, DBEM, DBPM, EcoOcean, EcoTroph, and FEISTY project negative amplification when SST increases by 1°C. Furthermore, the R ratio tend to reach asymptotic values at these high temperatures compared to GFDL forcing.

### 3.5 Propagation of trophic amplification across the food web

**Fig 8:**
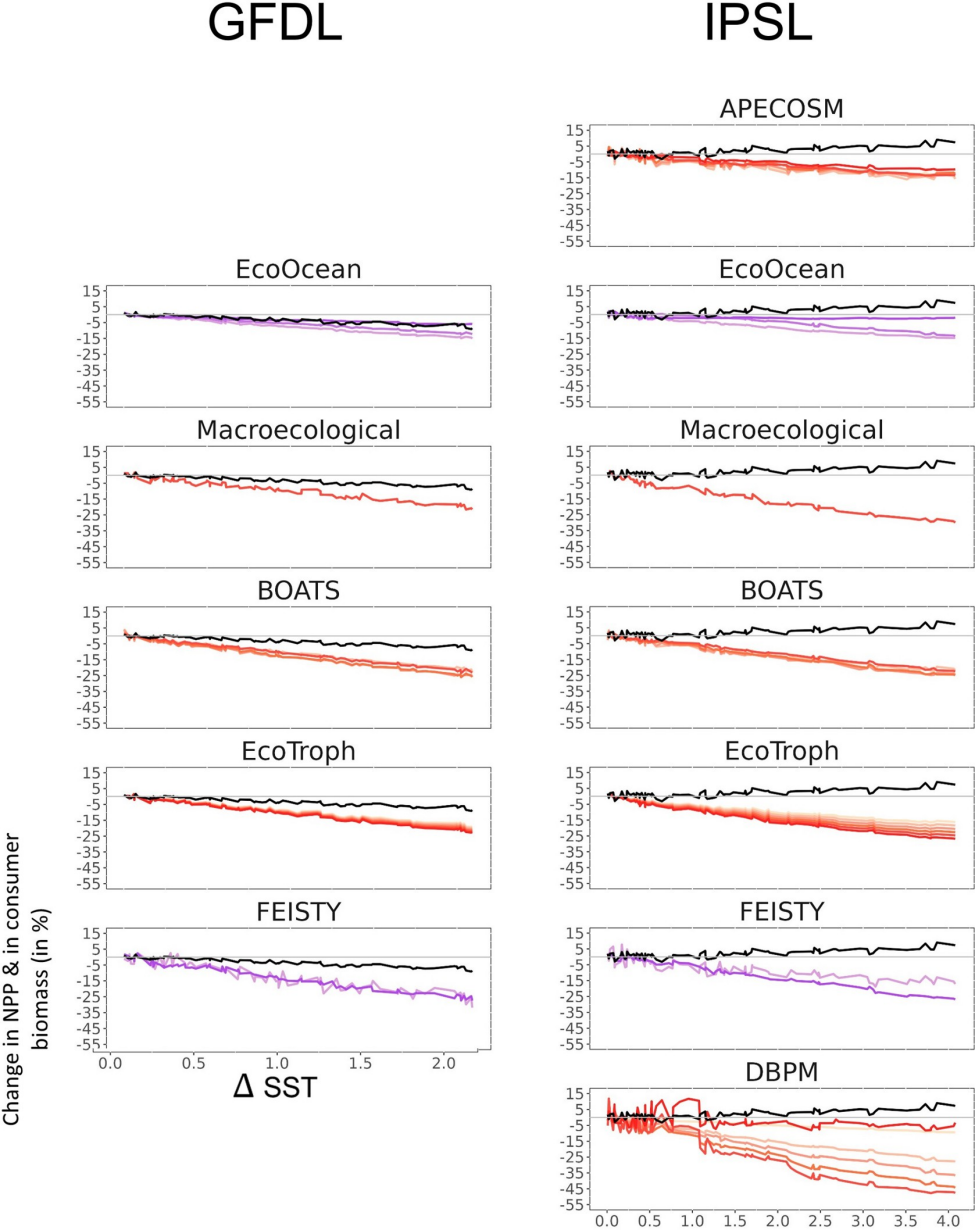
Disaggregation of consumer biomass response across the food web. The red gradient indicates changes in consumer biomass by weight classes, the purple gradient indicates changes in consumer biomass by length classes, and change in NPP is reported in black for the framework forced by GFLD (left column) and IPSL (right column) outputs under SSP5-8.5. Gradients light colours to dark colours correspond to small size classes to large size classes, respectively.

When disaggregating trophic amplification along the food web by size, MEMs exhibit different responses but an enhancement of observed patterns as SST increases (Fig 8). There is a dampening of the loss of consumer biomass between each size class of the food web for EcoOcean. While an amplification is projected for the smaller size classes, the magnitude of the decrease in biomass of individuals >90cm is similar to that of NPP under GFDL forcing. Under IPSL, all size classes show a negative inversion, but this inversion is larger for the small size class and close to zero for individuals >90cm. APECOSM exhibits similar patterns with less negative inversion toward the larger consumers. For BOATS, although the negative amplification (GFDL) or negative inversion (IPSL) with temperature across size classes is comparable, mid-size classes (100g-1kg, 1-10kg) show the largest response. Macroecological shows identical amplifications or inversions at all weight classes under GFDL and IPSL forcing respectively. EcoTroph and FEISTY show greater negative changes in biomass (amplification or inversion) with increasing size. DBPM exhibits an increase in the magnitude of negative inversion of consumer biomass with increasing size as EcoTroph and ZooMSS under IPSL, except the highest-size class (>100kg) has the smallest response.

## 4. Discussion

### 4.1 Various biomass responses to global warming

In response to climate change, NPP is projected to change. In the absence of any other modification of processes in the ecosystem, a similar change would be observed in the total consumer production and biomass. Nevertheless, the MEMs ensemble projections driven by GFDL suggest that a global negative trophic amplification, characterised by a decrease in total consumer biomass much larger than the decrease in NPP, is projected throughout the 21st century under a high emissions SSP5-8.5 scenario. In addition, the global increase in NPP projected by IPSL does not lead to a global increase in consumer biomass but a decrease that is even larger than for GFDL in some cases. These outcomes suggest the projected decrease in biomass may be at least partially independent of the change in NPP since it occurs even when a positive change in NPP is projected. The amplification of the input NPP signal, what is commonly called “trophic amplification” appears to be the result of additional processes leading to a decrease in consumer biomass. Thus, with or without NPP decrease, based on SSP5-8.5 GFDL and IPSL simulations, the average additional projected decrease in consumer biomass in the 2090s is on the same order of magnitude (16.7% ± 9.5%). All MEMs we considered are forced by temperature (SST, epipelagic temperature, and/or bottom temperature), primary producers (production/biomass depending on MEMs) and/or secondary producers (S1 Table and S9 Fig; [5]), which implies that the projected decrease in consumer biomass occurring throughout the food web is most likely induced, at least partly, directly or indirectly by temperature-related rates or processes.

Spatial variability of biomass responses was observed, highlighting different types of responses throughout the food web. On the one hand, where NPP is projected to decrease, 61.4% and 31.4% of ocean area under GFDL and IPSL, respectively (S3 and S5 Figs), a "negative amplification" response is projected by a majority of MEMs (89% and 82% of the ocean surface on average, respectively). Previous studies [22, 24] pointed out similar areal coverage of negative amplification at low trophic levels, but here we show that amplification has a similar areal coverage when considering the whole food web. In all MEMs, temperature seems to amplify the energy losses when the NPP is projected to decrease. On the other hand, where NPP is projected to increase, various responses are projected but the main response is an inversion of the climate signal from primary producers to higher trophic levels for all MEMs leading on average to “negative inversion” on 70% of the considered ocean surface, according to both IPSL and GFDL. This negative inversion also strengthens with increasing temperature.

### 4.2 Limitations of the model intercomparison

First, the use of two ESM forcings highlights one major caveat that is now well known: the uncertainties associated with the trends of NPP and lower trophic level response to climate change in the ESMs. Furthermore, while agreement between ESMs over SST has been improved from CMIP5 to CMIP6, the differences in projected NPP increased: NPP projections differ ±30% between CMIP5 and CMIP6 [5, 12]. Specifically in our study, the NPP trends over the late 21^st^ century are different under GFDL and IPSL (Figs 1B and S2B). Given the importance of NPP and low trophic levels on the functioning of ecosystems and their influence on the climate response projected by the MEMs [5, 18], there is a strong need for more research on their response to climate change to constrain uncertainty.

Secondly, to enable the best possible comparison between models, we do not consider fishing effects because not all Fish-MIP MEMs represent fishing and also because plausible future scenarios of fishing were not available. Future Fish-MIP simulations that include fishing may allow us to investigate the fishing effects on trophic amplification. By inducing additional biomass losses at mid- and high-trophic levels, fishing in the MEMs may exacerbate the projected trophic amplification, or alternatively cause a top-down trophic cascade of alternating amplification and attenuation or inversion (e.g., [50]).

Moreover, seven out of nine of the MEMs we considered are forced by temperature and primary and/or secondary producers [5, 35], without considering variables such as deoxygenation and acidification, which may act in combination to warming and magnify trophic amplification.

### 4.3 Various mechanisms towards consistent model responses?

As shown in S1 Table, all models considered in this study differ and the variability observed among MEM biomass response types reflects the different ways in which forcing variables (temperature and primary production/biomass or plankton concentration) can be integrated into MEMs with different fundamental assumptions [19].

Despite differences in the total consumer biomass responses to changes in NPP, six MEMs exhibit agreeing temporal and spatial patterns from the most complex one, APECOSM, to models of intermediate complexity, FEISTY, ZooMSS and BOATS, to the simplest ones, Macroecological and EcoTroph. Although there are many differences between these MEMs, our study suggested that temperature-driven processes may be pivotal drivers to explain trophic amplification patterns. While temperature has a direct and negative impact on biomass in EcoTroph and Macroecological, interactions of multiple temperature-dependent processes drive the responses of APECOSM, FEISTY, ZooMSS and BOATS. Within these four models, different rates such as growth, mortality and basal metabolism can be affected by temperature. Taken together, these processes generally cause a warming-induced decrease in biomass. For example, in FEISTY, basal metabolism increases faster than ingestion with temperature increases, so warming reduces the scope for growth, leading to a decrease in biomass [51]. In BOATS, warming influences the representative size of primary producers and sets the scope for growth of fish communities [48], warming causes an increase of the growth, reproduction and mortality rates leading at food web scale to a decrease in biomass [19, 37]. The differences in the processes which are affected by temperature may explain the differences in the biomass responses in terms of magnitude, type of response and spatial pattern. For instance, in APECOSM, the temperature-induced changes in growth and metabolic rates, which determine the changes in biomass, varies with food limitation (more important in limited food regions than in not limited food regions). These temperature-associated mechanisms may cause the observed negative amplification where NPP is projected to decrease while they lead to positive attenuation or positive inversion (depending on warming magnitude and associated impact) where NPP is projected to increase.

In contrast to the six aforementioned models, DBEM, DBPM and EcoOcean differ in terms of amplification (no amplification at global scale in DBEM and EcoOcean) and spatial pattern in biomass response to change in NPP (Figs 4 and S4). Their response to temperature changes may explain, at least partly, the absence of trophic amplification and their differences with other models. For example, in EcoOcean, environmental conditions and notably temperature drive habitat suitability which can cause animals to relocate, and some animals can move across several 1-degree grid cells in a month. Thus, observed changes in biomass in a grid cell are only partially attributable to trophic cascades, which makes it harder to pinpoint trophic amplification [40]. For DBEM, a mechanistic species distribution model, temperature affects production by altering the individual growth of each modelled species that subsequently affects its population dynamics. Temperature and other environmental variables drive the spatial distribution of species. However, in this model, organisms do not interact except with primary producers [19, 52]. This could be a potential reason for its weaker trophic amplification, supported by the fact that DBEM has the lowest or near lowest ratio values (Figs 6A and S7A). Specifically, NPP is a key parameter for the carrying capacity. As a result, warming-induced species shift from tropical area (biomass decreases and niche contraction) to higher latitudes and NPP decreases leads to negative amplification; In contrast, in higher latitude regions, the projected increases in NPP increase the carrying capacity of these areas while the expansion of species range from lower latitude region results in a positive relationship between NPP and upper trophic level biomass i.e “positive amplification”. In DBPM, growth and mortality rates scale with temperature at the same rate [39]. Therefore, temperature effects largely offset each other at the scale of the entire food web leading to an attenuation of the signal at the base of the food web.

### 4.4 Mechanisms resulting in trophic amplification propagation through the food web

Although we found a high degree of consistency between the MEMs when examining trophic amplification at the scale of total consumer biomass, breaking down the phenomenon at different size classes (indicative of trophic levels) for each MEM revealed different responses to warming waters. These responses can be partly explained by the representation of the structure and functioning of the food web in each MEM.

In EcoTroph, the projections show a continuous and progressive increase in magnitude of changes in biomass (either negative amplification or inversion) when moving up the food web. In this MEM, warming-induced changes in transfer efficiency result in a cumulative decrease in production from one trophic level to the next due to larger energy losses between each [53]. This reduction of the energy flux induces a progressive and continuous amplification of the negative responses with increasing consumer size [27]. The underlying ecological assumption is that, under ocean warming, species assemblages will change and become more and more dominated by species with lower transfer efficiency because of larger energy losses due to their metabolism processes that scale with temperature [18, 54, 55].

FEISTY projected a global amplification of biomass decreases from small (<30cm) to large organisms (>90cm). Metabolic demands increase with both temperature and size such that the reduction in the scope for growth with increasing temperature is worse for large, predatory fishes in FEISTY. This relationship drives the strong negative amplification and its propagation through the food web.

DBPM projections show a negative amplification of changes in biomass from small (1-10g) to large (10-100kg) organisms. This reflects the interplay of food and temperature dependent growth and mortality under a combination of warming and declines in phytoplankton at the base of the size-structured food-web. Warming increases feeding rates which translates into faster feeding and higher predation mortality rates, leaving less food available to maintain biomass of larger predators that feed on increasingly large prey. However, the largest organisms considered (>100kg) do not follow this pattern and show a similar change in biomass to that of the smallest organisms (1-10g). The biomass of these largest organisms is low compared to that of the other size classes due to the overriding effects of senescence mortality at very large sizes in the model.

On the contrary, APECOSM, BOATS, and Macroecological projections do not show different magnitudes of change between consumer classes since the multiple impacts of warming may either compensate for each other or be independent of size.

EcoOcean projected a global dampening of biomass decreases from small (<30cm) to large organisms (>90cm). This repression of climate signals could emerge from the interactions between faster reproducing but less mobile smaller organisms and slower reproducing but faster moving organisms. The resilience of the EcoOcean food web, with higher trophic level groups generally better able to relocate away from warming areas, dampens trophic amplification.

### 4.5 Analysing trophic amplification based on the change in NPP comparison with total consumer production

In our study, we focused on the changes in NPP relative to consumer biomass. Yet, the gross production of the living biomass is also a key parameter of ecosystem functioning and one of the main drivers of their resilience [28, 29]. In particular, fisheries sustainability relies on the harvested part of the gross production rather than the biomass. A few studies like Du Pontavice et al (2021) and Petrik et al (2020) have investigated this difference between production and biomass and showed a larger decrease in biomass than in production. BOATS, EcoTroph, and Macroecological, three of the MEMs considered in this study, introduce an explicit relationship between these two parameters. In EcoTroph, production and biomass are linked by the trophic flow kinetics, i.e. the speed of biomass transfers through the food web [56, 57], which accelerates with temperature therefore leading to a larger decrease in biomass than in production. In Macroecological, biomass at any given size corresponds to the ratio of total production at that size, by the mass-specific production rate [44], whereas in BOATS, biomass spectrum is estimated directly from fish production spectrum and mortality rate [37]. Under CMIP6, the additional biomass decrease between total consumer production and consumer biomass is about 5% and 15% under GFDL and IPSL, respectively, for both EcoTroph, FEISTY, and BOATS and about 15% and 5% under GFDL and IPSL, respectively for FEISTY (S8 Fig).

Thus, an approach based on the productivity of ecosystems and not their biomass results in less trophic amplification, which suggests a smaller impact on fisheries potential catches.

### 4.6 Low trophic level, key MEMs driver, estimation uncertainty and consequences

In our study, we find that depending on low trophic level forcing in each MEM (low trophic level biomass vs. low trophic level production), the magnitude of biomass changes relative to primary productivity is very different (S9 Fig), which is consistent with the results highlighted in Heneghan et al (2021). The magnitude of the biomass response of MEMs is, on average, larger for MEMs driven at low trophic level by production than those driven by biomass forcing (S1 Table and S9 Fig). The nature of this difference, thus, could firstly lie in the inconsistency that arises from 1-way forcing with biomass or production [19]. This could dampen the consumer decline when forcing with biomass. For example, the comparison of 1-way and 2-way forcing with APECOSM supports this assumption, although the small difference in terms of global biomass decline might not explain all [50].

This study highlighted the relative changes in consumer biomass compared to changes in NPP to investigate trophic amplification. Hence, we compared a production (NPP) to a biomass (consumer biomass). In a supplementary analysis, we explored the changes of low trophic level drivers of each MEM (other than NPP; i.e., phytoplankton and zooplankton biomass) which have a very different spatial structure and temporal trend (S9 Fig) compared to NPP [5]. At the global scale, low trophic level (*i*.*e*., phytoplankton and zooplankton) biomass is projected to decrease under IPSL while NPP is projected to increase.. One of the mechanisms driving this inversion (increase in NPP, decrease in phytoplankton biomass) is when temperature-driven phytoplankton metabolic costs exceed what can be met with the increase in NPP. Under GFDL, the decrease in phytoplankton and zooplankton biomass is projected to be slightly stronger than that of NPP.

As shown in S9 Fig, trophic amplification based on low trophic level driver of each MEMis reduced but consistent with to trophic amplification based on NPP under GFDL.On the contrary, the signal inversion between NPP and other MEMlow trophic level driver, under IPSL, lead to a different perception of trophic amplification (compared to trophic amplification relative to NPP) for APECOSM; EcoOcean, FEISTY and ZooMSS. The inversion in trend under IPSL (Fig 3, changes in consumer biomass compared to changes in NPP) becomes an amplification for the four aforementioned models when considering the changes in the individual model’s low trophic level drivers (S9 Fig).

## 5. Conclusion

The main response to the global decrease in NPP is a negative amplification, resulting in a more pronounced decrease in consumer biomass. Our study suggests that this response may be due to the way temperature influences different physiological and ecological processes in each ecosystem model. When an increase in NPP is projected, the temperature can offset the positive impact by attenuating or reversing the changes in biomass, leading to smaller increases or even decreases in biomass corresponding to positive attenuation or negative inversion respectively. Disaggregation of trophic amplification across different sizes within the food web highlighted a greater decrease in biomass of larger, high trophic levels on average. As a result, there are believable parts of the ocean for which we may see a large climate-induced decline in fishable biomass despite no change or increasing NPP.

## Supporting information

S1 Table A taxonomy of marine ecosystem models taking part in the Fish-MIP project (modified from (Heneghan et al., 2021; Lotze et al., 2019; Tittensor et al., 2021)). (TIF)

S2 Table Conversion between trophic levels-based and size-based bins. (TIF)

S1 Fig Spatial patterns of projected total consumer biomass relative changes in percent. Shown are global ensemble projections at a 1 × 1 degree resolution. mean 2090s individual model projections of total consumer biomass relative change over the reference period (1995_2014) under: (a) GFDL-SSP5-8.5; and (b) IPSL-SSP5-8.5.
(TIF)

S2 Fig Main outputs of the earth system models considered in the current study. mean changes in Sea surface temperature (a) and net primary production (b) in the 2090s relative to the reference period 1995_2014, under SSP5-8.5 for IPSL.
(TIF)

S3 Fig Percentage of surface covered by each total consumer biomass response type, for the seven considered MEMS, under GFDL-SSP5-8.5 modelling. (TIF)

S4 Fig (a) Spatial distribution of the total consumer biomass response types, for the nine considered MEMs, forced with IPSL-SSP5-8.5 modelling and (b) magnitude of the different types of change for the seven considered MEMS forced with IPSL-SSP5-8.5 Each response type is estimated at a 1 × 1 degree resolution, from the change in consumer biomass and NPP expected at the end of the century, relatively to the reference period.
(TIF)

S5 Fig The percentage of surface covered by each total consumer biomass response type, for the nine considered MEMS, under IPSL-SSP5-8.5 modelling. (TIF)

S6 Fig Model agreement on biomass response types projected in the 2090s, under IPSL-SSP5-8.5 configuration. Response types correspond to at least seven out of nine models in agreement for the coloured cells while grey cells indicate where less than seven models project the same type of response and white cells where no data were available. The percentage numbers in the pie chart correspond to the relative surface areas.
(TIF)

S7 Fig Evolution of R ratio (total consumer biomass change divided by NPP change) in regards to SST increase relative to reference period. Where NPP is expected to decrease (a), or increase (b) under IPSL-SSP5-8.5 combination. Grey lines separate biomass response types. (1), (2) and (3) refer to positive inversion, negative attenuation and negative amplification, respectively, while (4), (5) and (6) refer to positive inversion, negative attenuation and negative amplification, respectively.
(TIF)

S8 Fig Comparison between change over time in the total consumer production and change in total consumer biomass. Under EcoTroph simulation (first row) FEISTY simulation (second row) and BOATS simulation (third row). The Left and the right plots correspond to GFDL-SSP5-8.5 and IPSL-SSP5-8.5 forcing, respectively. Blue, and red lines correspond to the total consumer biomass change and total consumer production change, respectively.
(TIF)

S9 Fig Ensemble projections of low trophic level drivers change and total consumer biomass changes, relative to 1995–2014, under SSP5-8.5 and for all the considered MEMs with the GFDL-SSP5-8.5 forcing (a) and with the IPSL-SSP5-8.5 forcing (b). For temporal trends, all values are relative to the standardised reference period of 1995–2014. Vertical grey line indicates the last year of the historical period. Full lines correspond to MEMs’ total consumer biomass change and lines with diamonds to MEMs’ low trophic level changes.
(TIF)
