# Supplementary figures and images for: Trophic amplification: A model intercomparison of climate driven changes in marine food webs
Source: PLoS One. 2023 Aug 23;18(8):e0287570. doi: 10.1371/journal.pone.0287570 (PMC10446190; doi:10.1371/journal.pone.0287570)

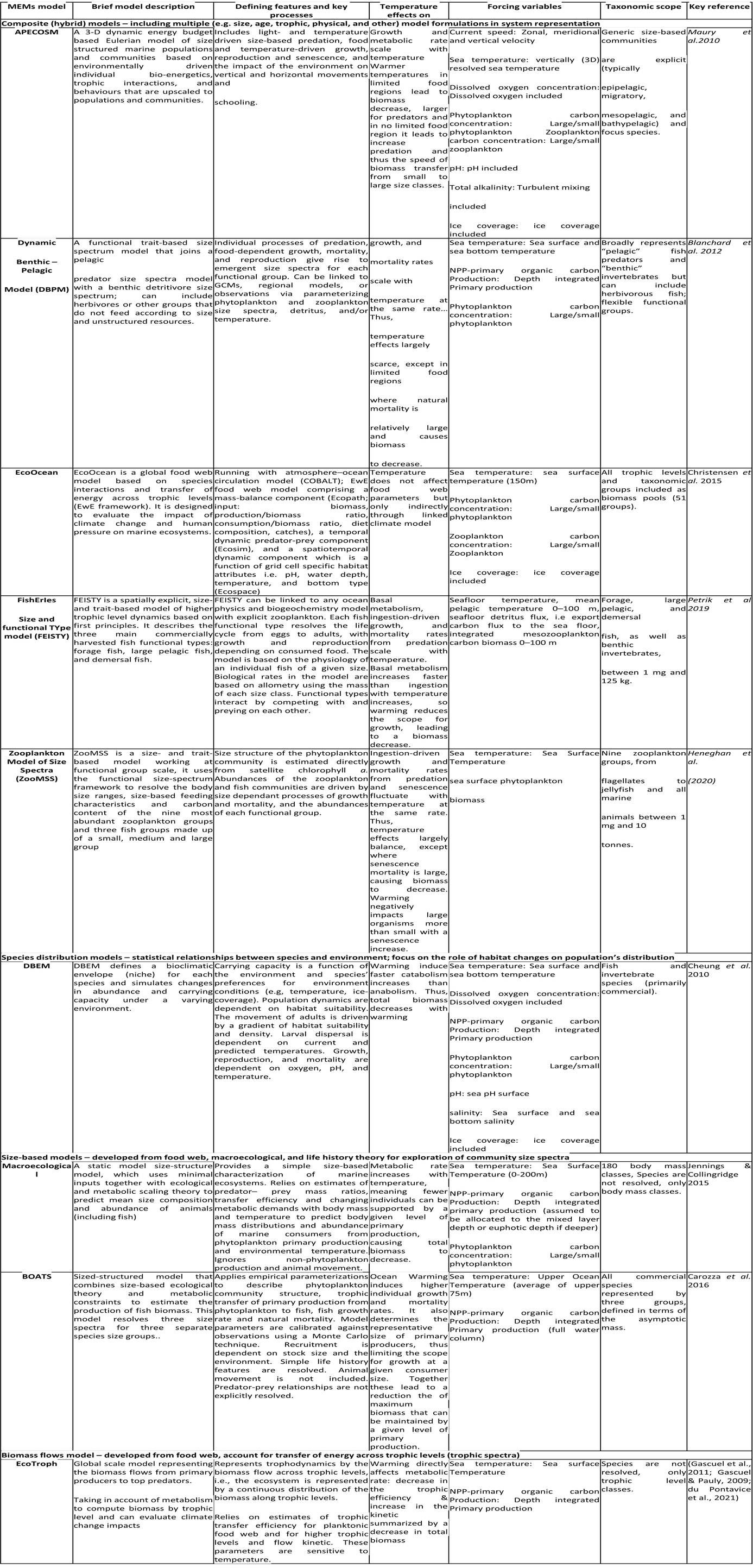

Supplement: S1 Table — (TIF) [file pone.0287570.s001.tif]

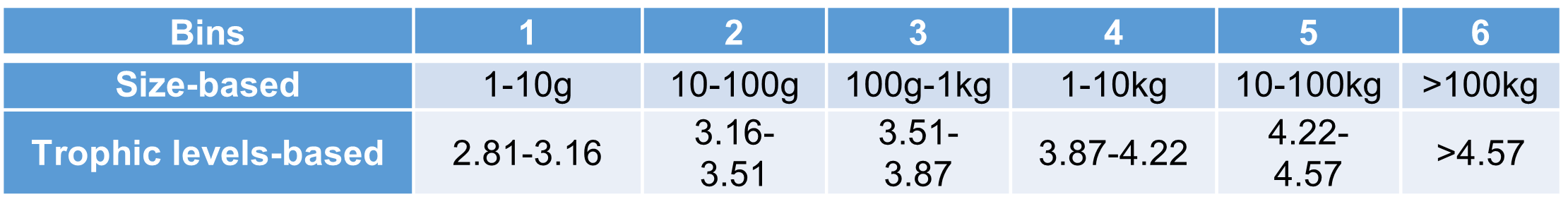

Supplement: S2 Table — (TIF) [file pone.0287570.s002.tif]

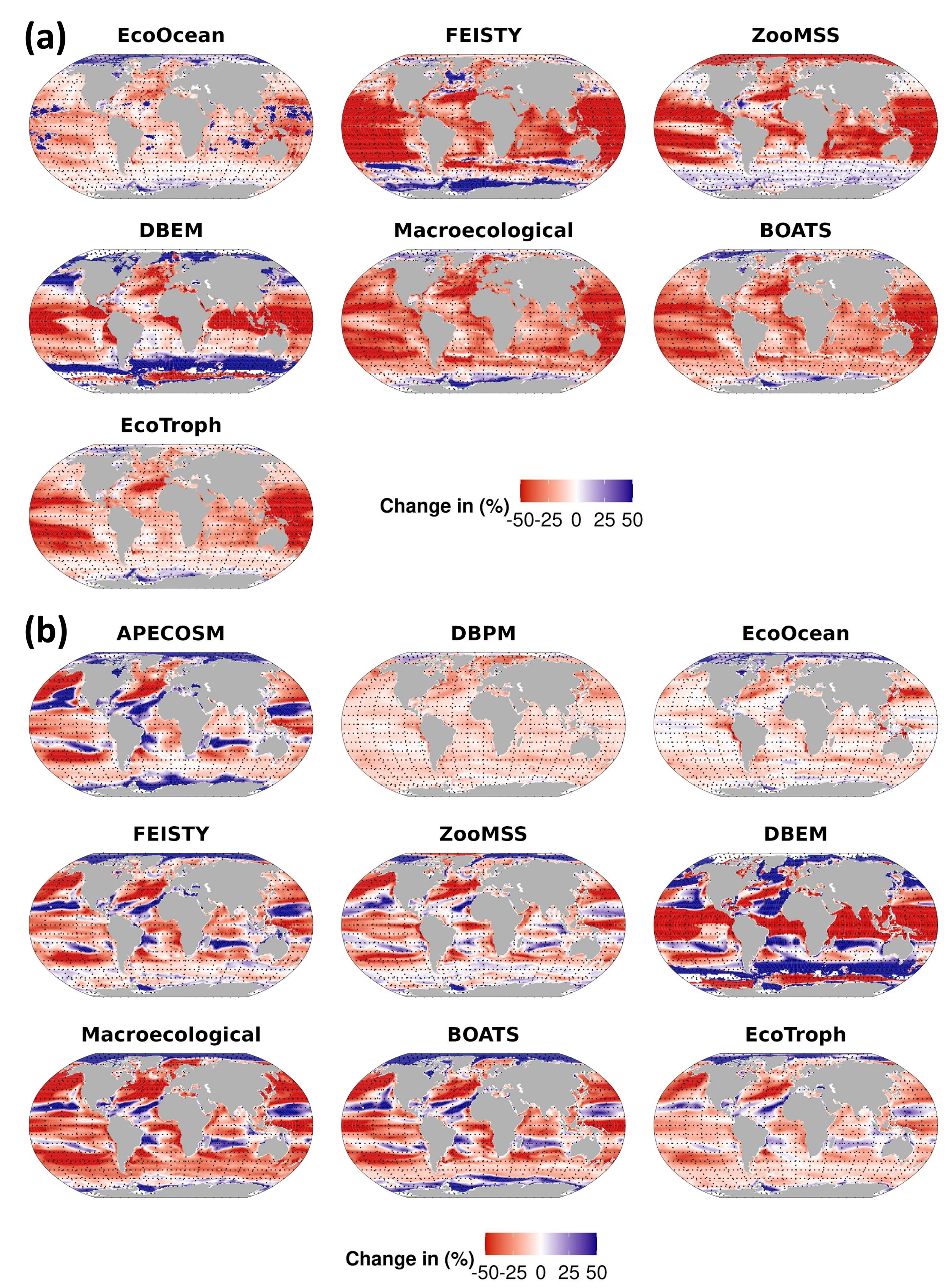

Supplement: S1 Fig — Shown are global ensemble projections at a 1 × 1 degree resolution. mean 2090s individual model projections of total consumer biomass relative change over the reference period (1995_2014) under: (a) GFDL-SSP5-8.5; and (b) IPSL-SSP5-8.5. (TIF) [file pone.0287570.s003.tif]

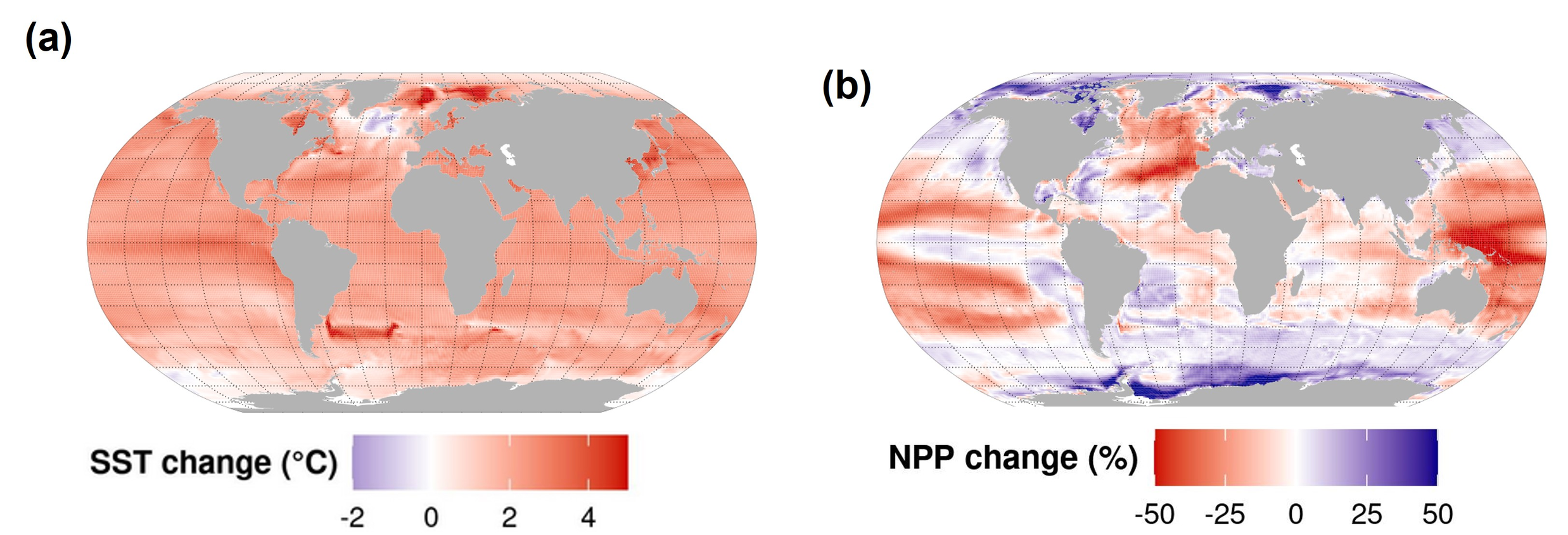

Supplement: S2 Fig — mean changes in Sea surface temperature (a) and net primary production (b) in the 2090s relative to the reference period 1995_2014, under SSP5-8.5 for IPSL. (TIF) [file pone.0287570.s004.tif]

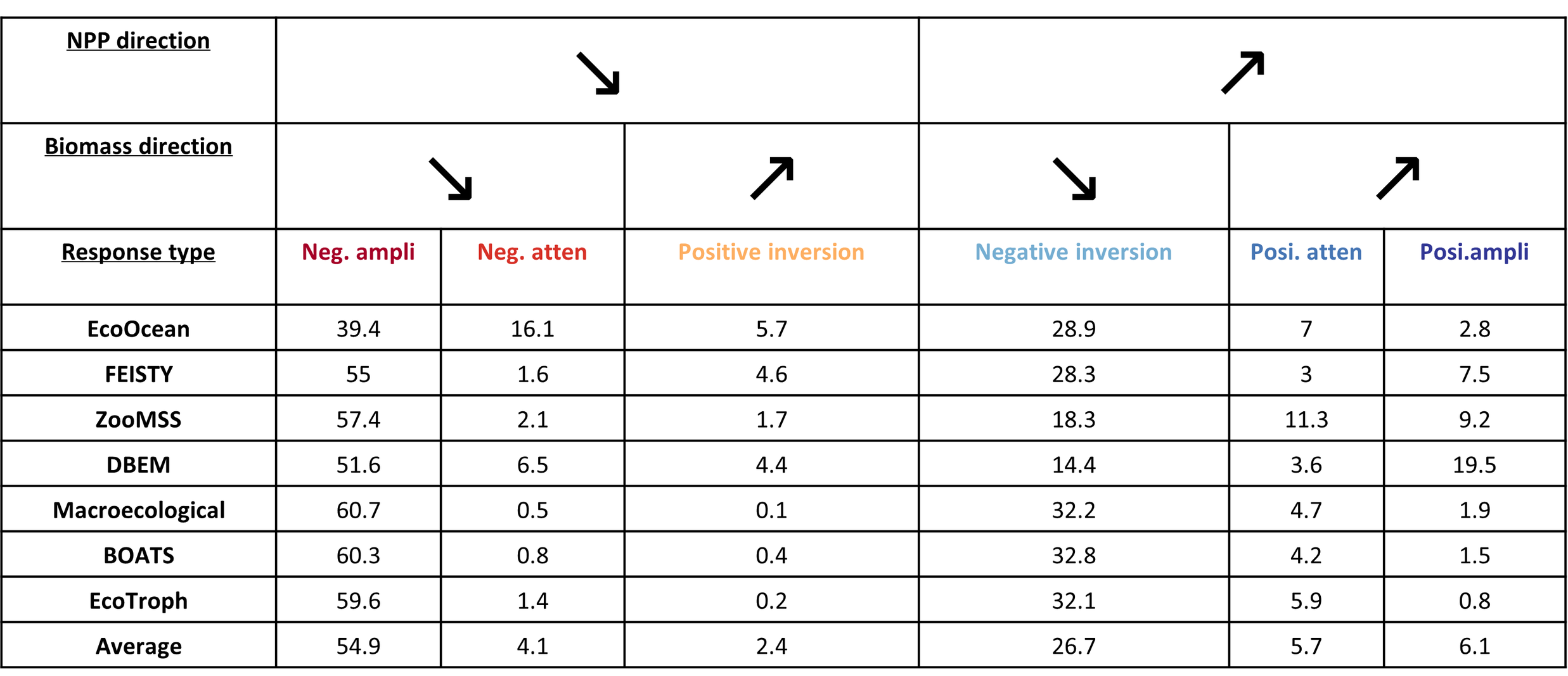

Supplement: S3 Fig — (TIF) [file pone.0287570.s005.tif]

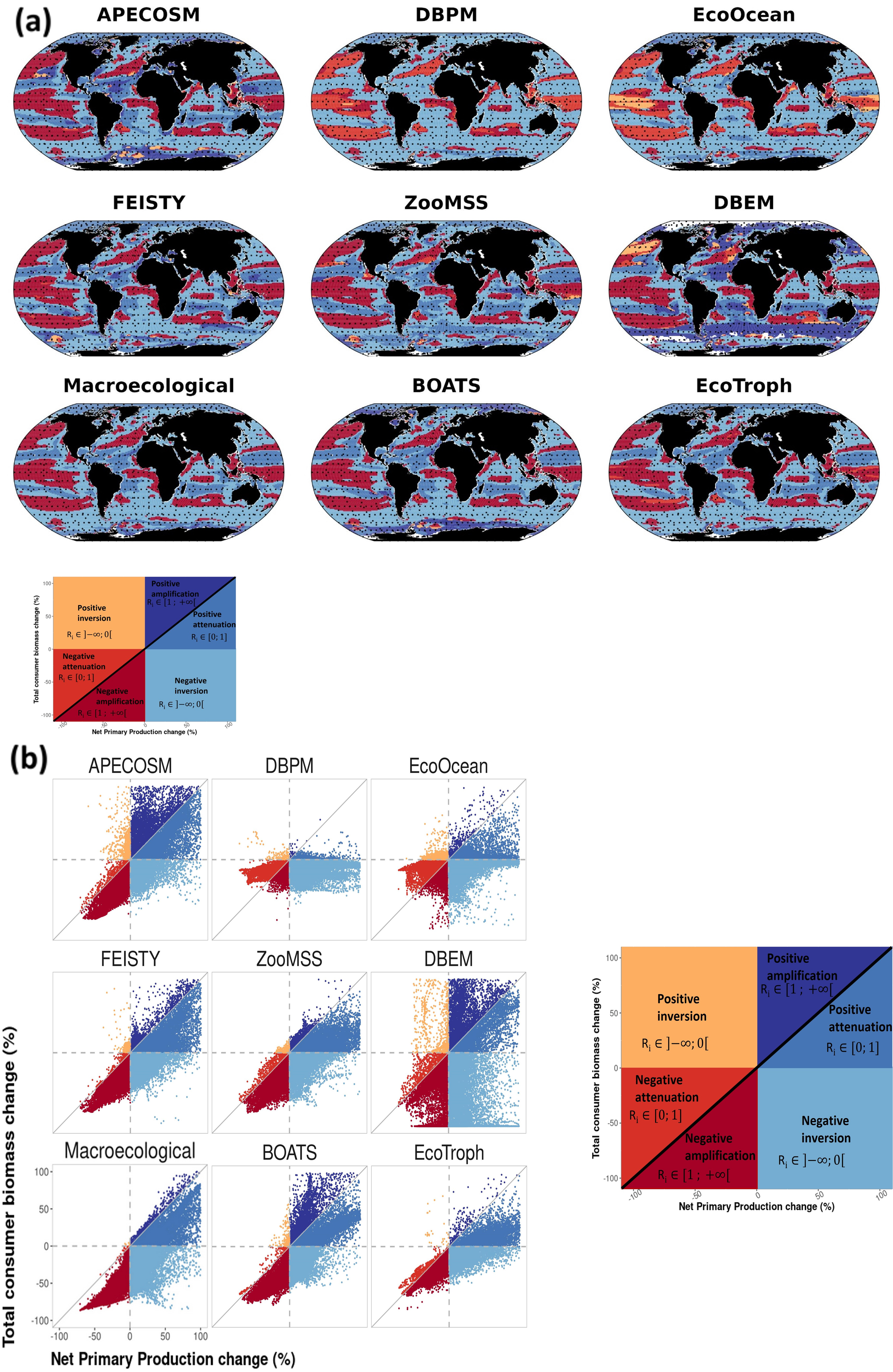

Supplement: S4 Fig — (a) Spatial distribution of the total consumer biomass response types, for the nine considered MEMs, forced with IPSL-SSP5-8.5 modelling and (b) magnitude of the different types of change for the seven considered MEMS forced with IPSL-SSP5-8.5 Each response type is estimated at a 1 × 1 degree resolution, from the change in consumer biomass and NPP expected at the end of the century, relatively to the reference period. (TIF) [file pone.0287570.s006.tif]

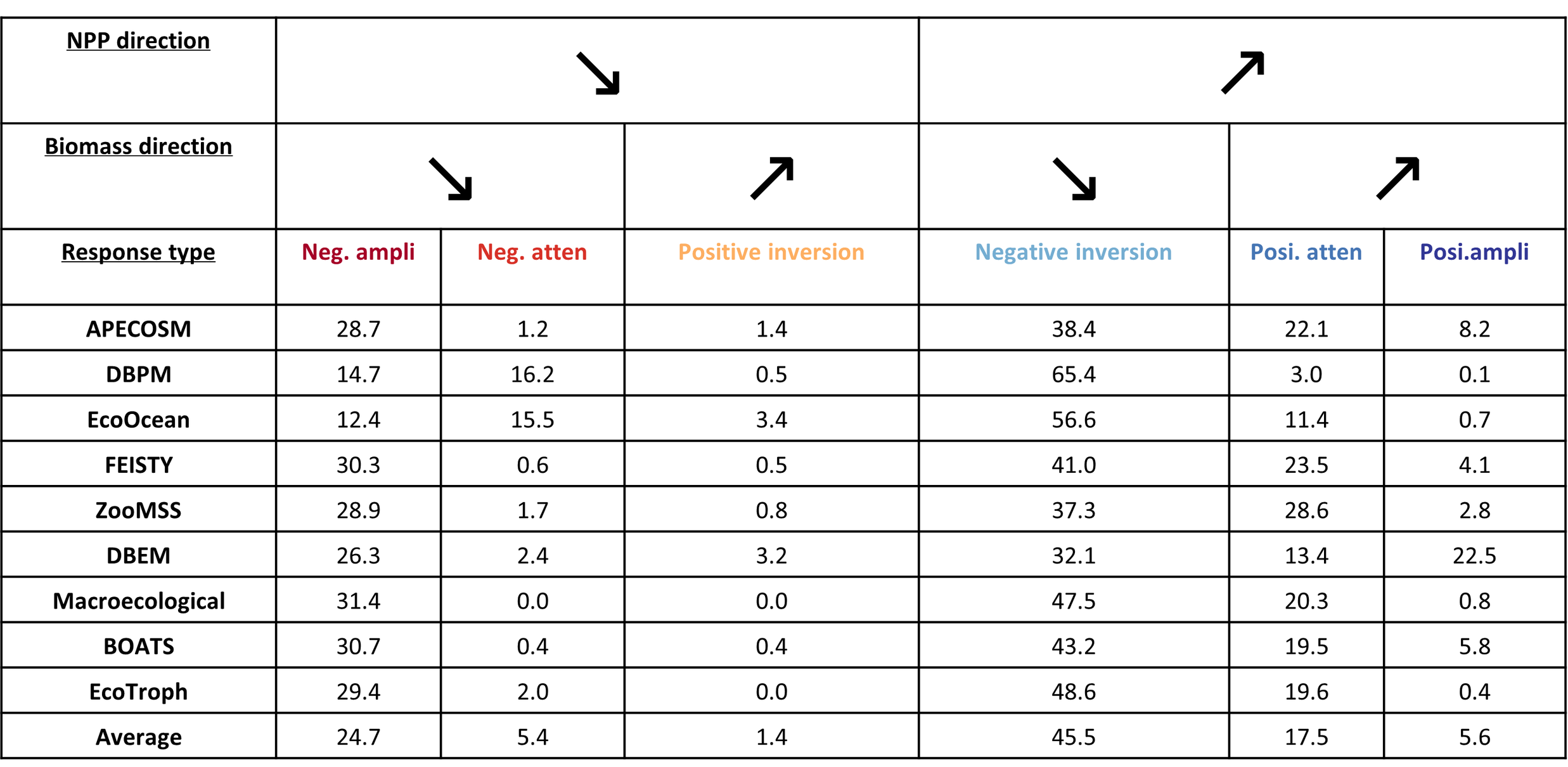

Supplement: S5 Fig — (TIF) [file pone.0287570.s007.tif]

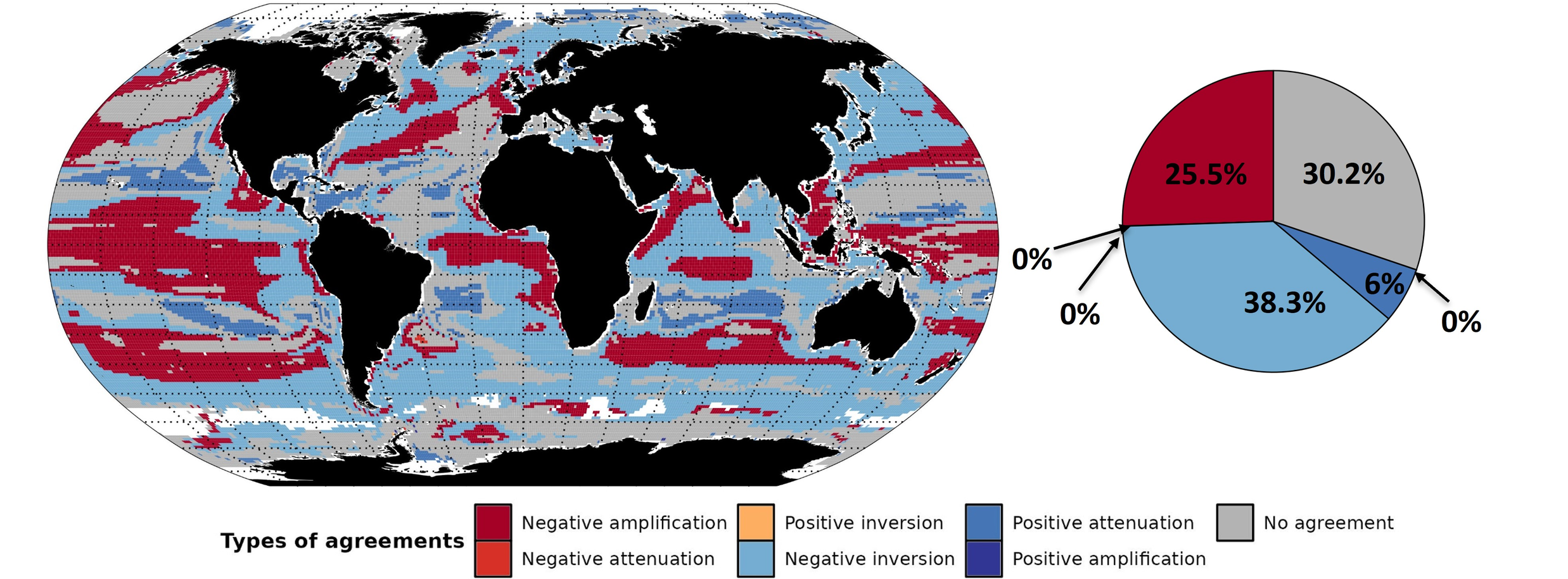

Supplement: S6 Fig — Response types correspond to at least seven out of nine models in agreement for the coloured cells while grey cells indicate where less than seven models project the same type of response and white cells where no data were available. The percentage numbers in the pie chart correspond to the relative surface areas. (TIF) [file pone.0287570.s008.tif]

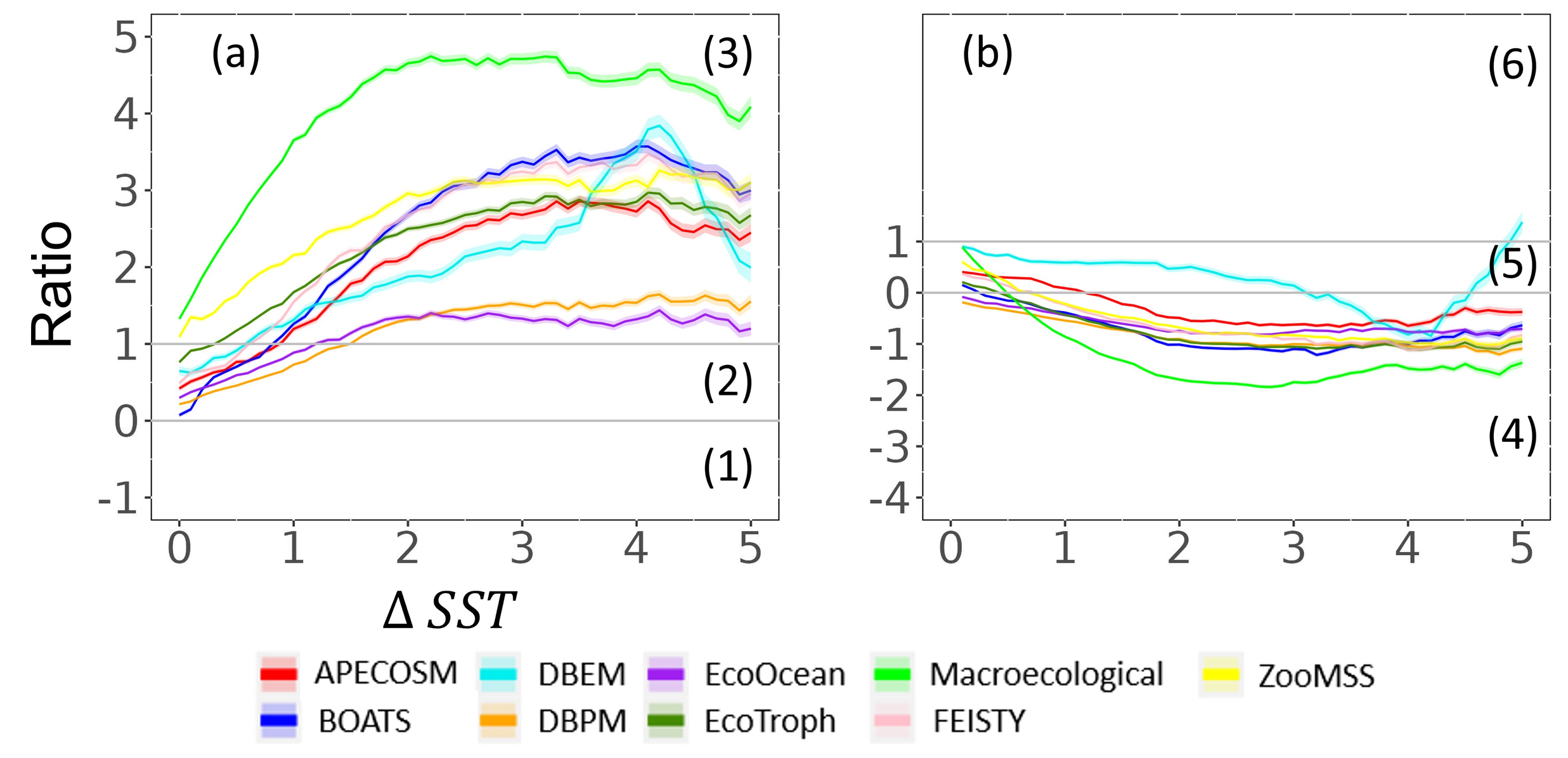

Supplement: S7 Fig — Where NPP is expected to decrease (a), or increase (b) under IPSL-SSP5-8.5 combination. Grey lines separate biomass response types. (1), (2) and (3) refer to positive inversion, negative attenuation and negative amplification, respectively, while (4), (5) and (6) refer to positive inversion, negative attenuation and negative amplification, respectively. (TIF) [file pone.0287570.s009.tif]

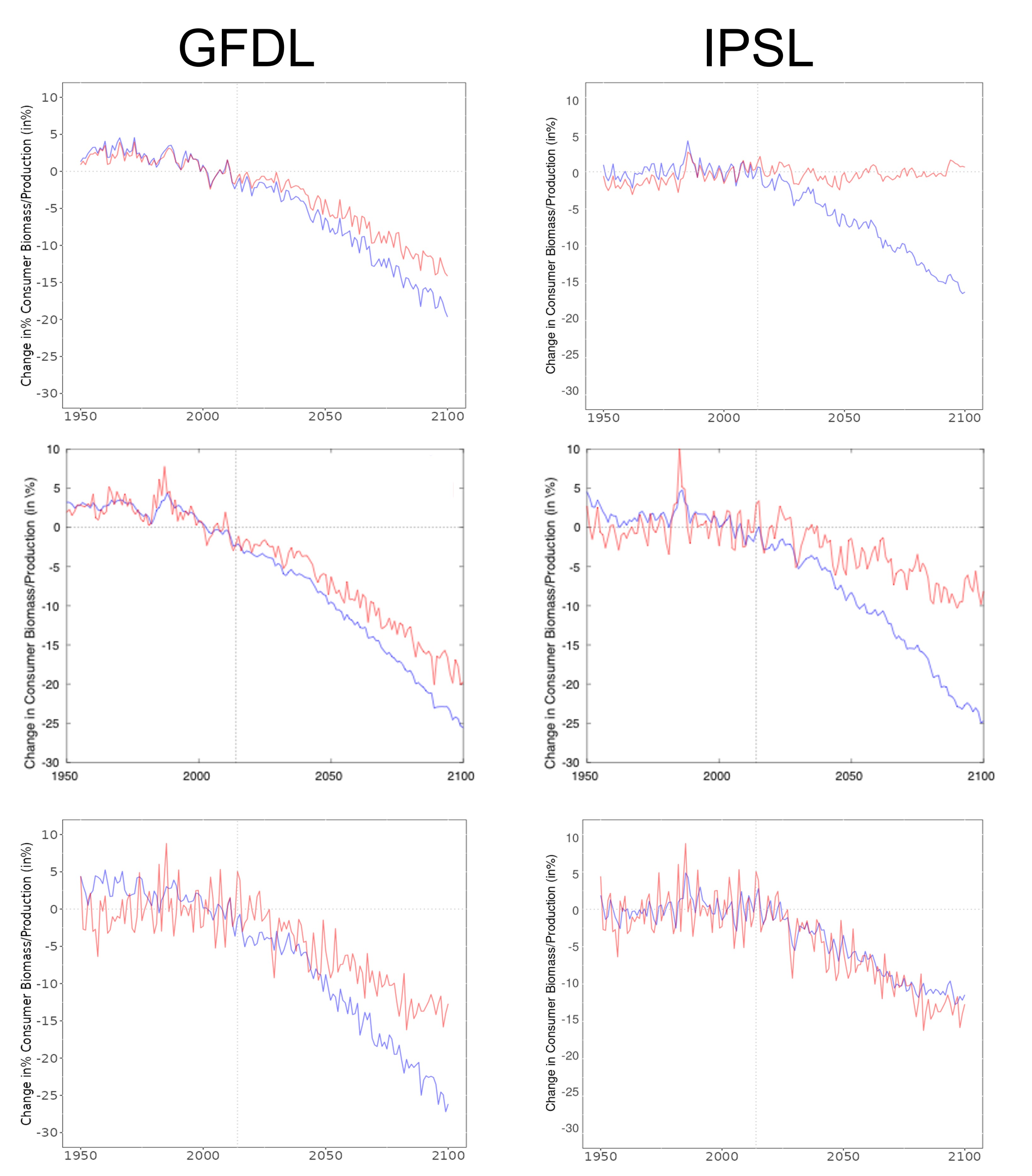

Supplement: S8 Fig — Under EcoTroph simulation (first row) FEISTY simulation (second row) and BOATS simulation (third row). The Left and the right plots correspond to GFDL-SSP5-8.5 and IPSL-SSP5-8.5 forcing, respectively. Blue, and red lines correspond to the total consumer biomass change and total consumer production change, respectively. (TIF) [file pone.0287570.s010.tif]

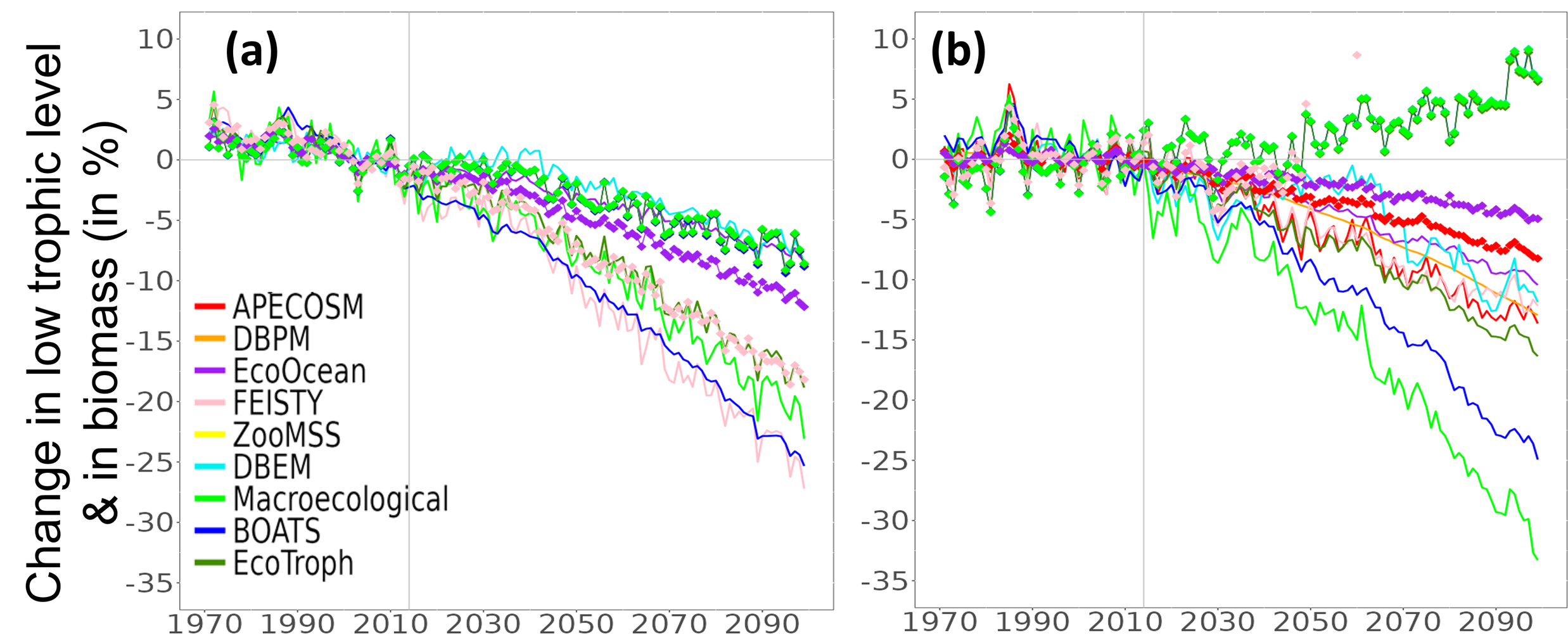

Supplement: S9 Fig — Ensemble projections of low trophic level drivers change and total consumer biomass changes, relative to 1995–2014, under SSP5-8.5 and for all the considered MEMs with the GFDL-SSP5-8.5 forcing (a) and with the IPSL-SSP5-8.5 forcing (b). For temporal trends, all values are relative to the standardised reference period of 1995–2014. Vertical grey line indicates the last year of the historical period. Full lines correspond to MEMs’ total consumer biomass change and lines with diamonds to MEMs’ low trophic level changes. (TIF) [file pone.0287570.s011.tif]
